# Fast-Dissolving Sodium Alginate-Based Microneedle Patch Integrating Tranilast and Glabridin-Loaded Nanoparticles for Dual-Action Hypertrophic Scar Therapy

**DOI:** 10.34133/bmr.0351

**Published:** 2026-04-13

**Authors:** Ayesha Younas, Muhammad Sohail, Yueting Li, Quan Zhou, Yonghui Shen, Shuanghu Wang, Yaru Shi, Zhenqiu Shang, Chuxiao Shao, Jian Xiao

**Affiliations:** ^1^ Key Laboratory of Joint Diagnosis and Treatment of Chronic Liver Disease and Liver Cancer of Lishui, Central Laboratory of The Lishui Hospital of Wenzhou Medical University, The First Affiliated Hospital of Lishui University, Lishui People’s Hospital, Lishui, Zhejiang 323000, China.; ^2^ School of Pharmaceutical Sciences, Wenzhou Medical University, Wenzhou, Zhejiang 325000, China.; ^3^ Department of Wound Healing, The First Affiliated Hospital of Wenzhou Medical University, Wenzhou 325035, China.; ^4^ Zhejiang Key Laboratory of Imaging and Interventional Medicine, Zhejiang Engineering Research Center of Interventional Medicine Engineering and Biotechnology, Lishui Hospital of Zhejiang University, Lishui, Zhejiang 323000, China.; ^5^ Department of Wound Repair and Regenerative Surgery, The First Affiliated Hospital of Lishui University, Lishui People’s Hospital, Lishui, Zhejiang 323000, China.

## Abstract

Hypertrophic scars (HSs) are a prevalent skin condition that imposes substantial functional, financial, and aesthetic burdens, with existing treatments often leading to recurrence, pain, and adverse effects. To address these issues, we propose a first-in-class fast-dissolving sodium alginate-based microneedle patch (sMN) that achieves sequential, dual-release kinetics by integrating tranilast (TS) and glabridin (GL)-loaded nanoparticles (GLNPs), termed TS+GLNPs@sMN. Our system uniquely integrates rapid TS release for immediate “OFF” switching, suppression of fibroblast activation, stabilization of mast cells, and relief of pruritus, with sustained GL release from GLNPs for prolonged “ON” switching, which promotes fibroblast apoptosis and modulates scar matrix deposition. This sequential “OFF-to-ON” paradigm mirrors the temporal progression of HS pathophysiology, with early inflammatory events preceding fibrotic remodeling. The cone-shaped microneedle patch (~565 μm) demonstrated efficient skin penetration/recovery, even drug distribution, fast dissolution (180 ± 20 s), high loading capacity, and desirable release kinetics. In vitro evaluations confirmed promising anti-proliferative, anti-migratory, and anti-angiogenic effects. Preliminary in vivo biocompatibility in mice was satisfactory, with no skin irritation and normal complete blood count, renal, and hepatic function. In vivo studies in a rabbit ear HS model demonstrate accelerated HS healing, reduced HS thickness, epidermal thickness index (ETI), scar elevation index (SEI), and collagen deposition, along with decreased profibrotic markers, inflammatory cytokines, and angiogenic factors. Overall, the TS+GLNPs@sMN patch offers a minimally invasive, self-administrable, dual-action therapeutic strategy for enhanced HS management, with safety and efficacy in preclinical models.

## Introduction

Hypertrophic scar (HS) is a complex and globally prevalent skin condition, particularly in developed countries, affecting approximately 100 million individuals. This condition imposes considerable functional, financial, and aesthetic burdens on patients, often leading to substantial discomfort, pain, and pruritus [1,2]. HS is characterized by firm, red, elevated lesions, with associated symptoms such as bulging, flushing, and crispation. Thickened dermal and epidermal layers and a dysfunctional stratum corneum mark these lesions. The pathogenesis of HS is multifactorial, typically driven by a prolonged inflammation, excessive angiogenesis, and abnormal proliferation of dermal fibroblasts. This process is further complicated by the accumulation of myofibroblasts and collagen, irregularities in the extracellular matrix (ECM), and dysregulation of cytokine and growth factor signaling during the skin healing process [3–5]. Current treatments for HS include glucocorticoid injections or creams, surgical excision, laser therapy, radiotherapy, silicon sheeting, pressure dressings, and cryotherapy. However, these therapies often face limitations in efficacy, high recurrence rates, and a range of side effects, highlighting the need for novel therapeutic approaches [6,7].

Tranilast, *N*-(3′,4′-dimethoxycinnamoyl) anthranilic acid (*N*-5′) (TS), an analog of a tryptophan metabolite, exhibits a wide range of therapeutic effects, including anti-allergic, anti-proliferative, anti-inflammatory, anti-angiogenic, and cardioprotective [8]. It has been used to treat various conditions, including bronchial asthma, neuropathic pain, scleroderma, atopic dermatitis, keloids, HS, and allergic conjunctivitis, among others. Recent studies have shown that TS inhibits the NLRP3 inflammasome by binding to the NACHT domain, thereby preventing NLRP3 oligomerization [9,10]. This mechanism underpins its anti-inflammatory effects and contributions to improved cellular homeostasis. Additionally, TS suppresses mast cell mediators, reduces collagen deposition, inhibits transforming growth factor-β1 (TGF-β1) and α-smooth muscle actin (α-SMA), and regulates matrix metalloproteinase (MMP) secretion. These mechanisms enhance its effectiveness in treating HS and mitigating associated symptoms, such as itching [8,10,11]. However, the therapeutic potential of TS is constrained by its poor water solubility and bioavailability. Therefore, we selected its water-soluble sodium salt to improve formulation development.

*Glycyrrhiza glabra* (licorice), a traditional medicinal plant, contains over 300 bioactive compounds. Among these, glabridin (GL), a prenylated isoflavonoid, has garnered marked attention due to its diverse pharmacological profile [12]. GL exhibits a range of therapeutic properties, including antibacterial, antifibrotic, anti-inflammatory, and antiproliferative. Additionally, its multimodal mechanism includes pro-apoptotic, reduced collagen deposition, anti-angiogenic, and antioxidant activities [13–16]. The anti-fibrotic action of GL has been particularly well-documented, with studies demonstrating its ability to modulate several key molecular pathways. Specifically, GL inhibits the TGF-β1/SMAD signaling pathway, a crucial regulator of collagen synthesis and fibroblast activation. It also disrupts the FAK–Src complex, leading to reduced ECM production and improved collagen fiber organization. Moreover, GL modulates the phosphatidylinositol 3-kinase (PI3K)/AKT pathway to promote fibroblast apoptosis, thereby reducing scar formation [17,18]. Despite its therapeutic potential, GL’s clinical application is hampered by its hydrophobic nature, which results in poor aqueous solubility, low skin permeation, rapid clearance, and instability.

To overcome these challenges and enhance the therapeutic potential of GL, we have developed GL-loaded poly (d, l-lactide-co-glycolide) (PLGA) nanoparticles (GLNPs). PLGA, a well-established biocompatible and biodegradable copolymer composed of d, l-lactide and glycolide units, is renowned for its ability to provide controlled and sustained drug release [19,20]. PLGA nanoparticles (NPs) can serve as a protective carrier for GL, shielding the drug from premature degradation, improving aqueous solubility, and enabling site-specific controlled release. This formulation strategy leverages nanomedicine principles to enhance therapeutic efficacy through targeted delivery and controlled drug release [21], thereby maximizing the therapeutic benefits of GL while minimizing potential side effects.

HS often exhibits low permeability, a consequence of excessive fibroblast accumulation, which hinders the effectiveness of treatment strategies [22]. To overcome this challenge, microneedles have emerged as an effective transdermal delivery system, offering a means to bypass the skin barrier while combining the benefits of dressings and intradermal injections [23]. By micro-puncturing the stratum corneum, microneedles enable controlled, localized, and sustained drug release, reducing invasiveness compared to conventional methods [2]. Furthermore, microneedles exert a mechanical effect by disrupting collagen fibers, relieving mechanical stress in scar tissue, facilitating tissue remodeling, and inhibiting scar formation [24]. Among the different types of microneedles, dissolving microneedles made from biodegradable polymers stand out for their superior performance. These microneedles dissolve upon application, facilitating direct drug delivery to the target tissue. Their key advantages include biocompatibility, biodegradability, good drug loading capacity, and a simplified one-step application process, making them a practical and patient-friendly solution for skin-related treatments.

Sodium alginate, a linear anionic polysaccharide composed of β-d-mannuronic acid (M) and α-l-guluronic acid (G) derived from brown seaweed, exhibits excellent biocompatibility, high water absorbency, and ease of gelation [25]. Its film-forming ability [26] and suitability for microneedle preparation [27] make it a promising candidate for scar management and other dermatological treatments. In addition, sodium alginate possesses moisturizing, wound-healing, antimicrobial, anti-inflammatory, and potential anticancer properties, further broadening its biomedical potential [28]. Therefore, a sodium alginate-based microneedle system can not only reduce HS surface tension but also enhance skin hydration and elasticity, thereby softening the typically stiff scar tissue. Furthermore, to ensure effective HS treatment, microneedle patches require a flexible, detachable, and protective backing layer. Therefore, we selected polyvinylpyrrolidone (PVP) as the base layer to achieve these characteristics.

**Fig. 1.**
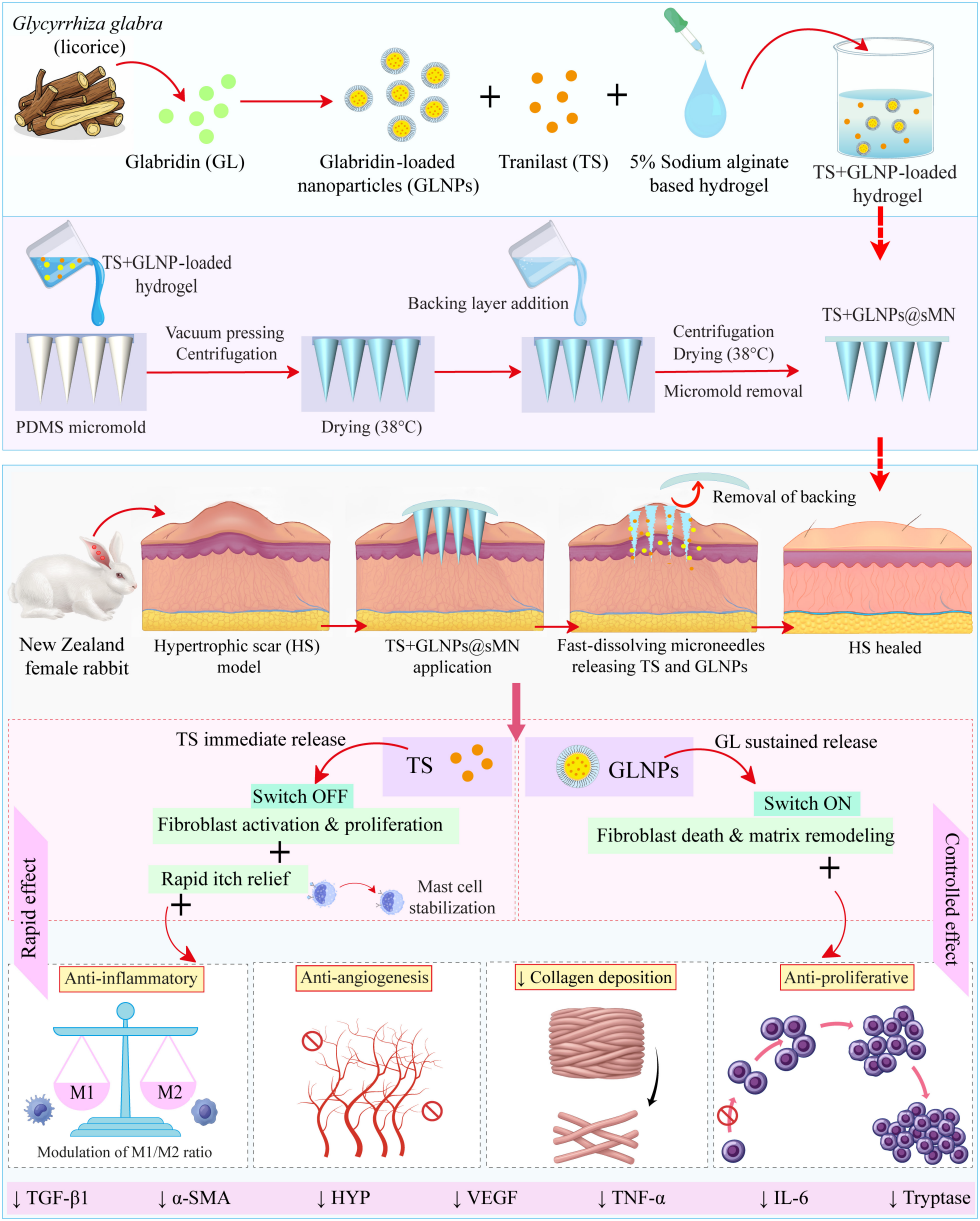
Schematic representation of the fabrication and application of the TS+GLNPs@sMN patch on an HS model, illustrating the sequential release of tranilast (TS) and glabridin (GL) for the synergistic HS therapy.

Herein, we propose a novel, biocompatible, fast-dissolving sodium alginate-based microneedle patch (sMN) engineered to incorporate TS and GLNPs (TS+GLNPs@sMN) for enhanced HS therapy (Fig. 1). This TS+GLNPs@sMN offers a single-dose, efficient drug delivery solution with sequential drug release kinetics. TS is incorporated into the microneedle patch to provide rapid therapeutic effects. By “switching OFF” fibroblast activation and proliferation, TS rapidly modulates the initial stages of scarring. It also offers fast-acting itch relief through mast cell stabilization and anti-inflammatory effects. GLNPs, on the other hand, were engineered to deliver sustained therapeutic benefits via controlled GL release. This sustained release “switches ON” fibroblast apoptosis and initiates matrix modulation via anti-inflammatory, anti-angiogenic, anti-proliferative, and collagen-degrading effects. Therefore, we hypothesize that this dual-action microneedle therapy will effectively address the complex pathophysiology of HS and demonstrate greater efficacy than conventional treatments. By offering a patient-friendly, minimally invasive modality for sustained treatment, this strategy can address critical barriers to long-term adherence and clinical efficacy.

## Materials and Methods

### Materials

#### Experimental materials

GL (C_20_H_20_O_4_) [purity ≥99%, molecular weight (Mw): 324.37 g/mol], PLGA [lactide:glycolide 50:50, [C_3_H_4_O_2_]x[C_2_H_2_O_2_]y, ester terminated, Mw: 7,000 to 17,000 g/mol], sodium alginate [(C_6_H_7_O_6_Na)_n_, purity ≥98%, Mw: 100,000 Da, viscosity 200 ± 20 mPa.s), and PVP (Mw: 58,000 g/mol) were obtained from Aladdin (Shanghai, China). Tranilast sodium (C_18_H_16_NNaO_5_) (purity 98%, Mw: 349.31 g/mol) was purchased from Macklin Biochemical Co. Ltd. (Shanghai, China). Rhodamine B (Rh.B) (C_28_H_31_ClN_2_O_3_) (Mw: 479 g/mol) was obtained from Solarbio Science & Technology Co. Ltd. (Beijing, China). Human HS-derived fibroblasts (HSFs) were sourced from Shanghai Baili Biotechnology Co. Ltd. (Shanghai, China). Dulbecco’s modified Eagle’s medium Nutrient Mixture F-12 (DMEM/F-12) (1:1), fetal bovine serum (FBS), and penicillin–streptomycin were procured from Gibco (Thermo Fisher Scientific, Shanghai, China). Phosphate-buffered saline (PBS) was purchased from Sigma-Aldrich Co. (St. Louis, MO, USA). Cell Counting Kit-8 (CCK-8) assay kit and calcein-AM/propidium iodide (PI) double staining kit were purchased from Dojindo Laboratories (Shanghai, China). Pentobarbital sodium (C_11_H_17_N_2_NaO_3_) (Mw: 248.26 g/mol) was obtained from Sinopharm Chemical Reagent (Shanghai, China). The rabbit TGF-β1 ELISA kit was obtained from Shanghai Yuanju Biotechnology Center (Shanghai, China). The hydroxyproline (HYP) colorimetric assay kit was purchased from Elabscience Biotechnology (Wuhan, China). The rabbit vascular endothelial growth factor (VEGF), tumor necrosis factor-α (TNF-α), and interleukin-6 (IL-6) enzyme-linked immunosorbent assay (ELISA) kits were sourced from YOBIBIO Biotechnology Co. Ltd. (Shanghai, China). Throughout this research study, reverse osmosis (RO) pure water (<5 μs/cm) or ultrapure water (18.2 MΩ.cm) (HyperPureX model: SD-20, Integrated water purification system, China) was used. All solvents used were high-performance liquid chromatography (HPLC)-grade.

#### Experimental animals

Male BALB/c mice (8 weeks; 18 to 20 g) and male C57BL/6 mice (6 weeks; 20 to 22 g) were obtained from SBF Biotechnology Co. Ltd. [Beijing, China; license SCXK (Beijing) 2024-0001]. Female New Zealand white rabbits (2.0 to 2.5 kg) were purchased from Pizhou Dongfang Animal Center (Pizhou, China). All animals were housed individually under specific pathogen-free conditions at 25 ± 2 °C and 55 ± 2% relative humidity (RH) on a 12-h light/12-h dark cycle. Mice were acclimated for at least 3 d, whereas rabbits underwent a 7-d acclimatization period with ad libitum access to irradiated chow and autoclaved water. All experimental procedures were approved by the Animal Ethics Committee of Lishui University (protocol no. 2025D009) and conducted in accordance with the National Research Council Guide for the Care and Use of Laboratory Animals.

### Fabrication of GLNPs

The GLNPs were synthesized using a modified single-emulsion solvent-evaporation method [29]. GL (20 mg) and PLGA (200 mg) were co-dissolved in 5 ml of dichloromethane (DCM) to form the organic phase. This solution was dropwise-injected through a 23-gauge needle into 20 ml of ice-cold 1% polyvinyl alcohol (PVA) solution. Sonication, with dropwise addition, was performed using a micro-tip probe (Φ2 mm) on an Ultrasonic Homogenizer (HX-ULTRASONIC, JY88-IIN, Shanghai Huxi Industrial Co. Ltd., China) set at 10 s “ON”/5 s “OFF”, 130 W, 60% amplitude, for 5 min at 4 °C to form an oil-in-water nanoemulsion (Fig. S1). Emulsification was immediately followed by magnetic stirring (400 rpm, room temperature, 12 h, in the dark) to allow complete DCM evaporation and NP solidification. The resulting NP suspension was ultracentrifuged (12,000*g*, 20 min, 4 °C) (TGL-18M, Benchtop High Speed Refrigerated Centrifuge, Chongqing, China) and washed twice with ultrapure water to remove residual PVA and unencapsulated GL. The suspension was then snap-frozen in liquid nitrogen and lyophilized for 48 h (−50 °C, 0.05 mbar, and under light-free conditions). The resulting dry powder was stored at −20 °C and used either as such (solid state) or reconstituted in ultrapure water immediately before use.

### Characterization of NPs

The freshly prepared NPs were diluted 1:20 (v/v) with ultrapure water and analyzed at 25 °C in disposable capillary cells. Hydrodynamic diameter (*Z* average), polydispersity index (PDI), and zeta-potential were recorded on a Litesizer 500 particle analyzer (Anton Paar, Austria). Surface morphology was examined by scanning electron microscopy (SEM; SU8600, Hitachi High-Tech Corp., Ibaraki, Japan). A drop of diluted dispersion was placed on a silicon wafer, air-dried, sputter-coated with a 5-nm gold layer under vacuum, and imaged at 5 kV. Additionally, the internal structure and surface morphology of the NPs were further investigated using transmission electron microscopy (TEM; HT7800, Hitachi High-Tech Corp., Japan), which provided detailed images revealing the core–shell architecture and confirming the uniform encapsulation of GL within the PLGA matrix. Moreover, the freshly prepared GLNPs were centrifuged at 12,000*g* for 20 min at 4 °C. The supernatant was filtered (0.22 μm, nylon), and the concentration of non-encapsulated GL was detected by HPLC with ultraviolet (UV) detection (HPLC-UV) (Agilent 1260 Infinity II HPLC with VWD G7114A UV detector). The pellet was lyophilized, and the total weight of recovered GLNPs was recorded. Further, the percentage encapsulation efficiency (EE %), percentage drug loading % (DL %), and percentage yield (Yield %) were calculated using the following equations:

$$EE\%=\left(\frac{\text{Total}\;GL-\text{Free}\;GL}{\text{Total}\;GL}\right)\times 100$$ (1)

$$\;DL\%=\left(\frac{\text{Total amount of encapsulated}\;GL}{\text{Total weight of}\;NPs}\right)\times 100$$ (2)

$$\begin{array}{c} \begin{array}{c} \text{Yield}\%=\left(\frac{\text{Total theoretical weight of GLNP component}\text{s}}{\text{Total recovered GLNP}s\;\text{after lyophilization}}\right)\times 100 \end{array} \end{array}$$ (3)

The chemical composition and functional groups of PLGA, GL, PVA, NPs, and GLNPs were characterized using Fourier transform infrared spectroscopy (FTIR) (Nicolet iS20 spectrometer, Thermo Scientific, USA). The spectra were recorded over the wavenumber range of 4,000 to 400 cm^−1^ using the KBr disc method. Samples were gently ground with dry KBr (1:100, w/w) and pressed under 10 t for 30 s to obtain transparent pellets for further analysis.

### In vitro drug release study of NPs

Dialysis method (under sink condition) was employed to quantify the extended release of GL from GLNPs versus free drug. Release medium (0.01 M PBS, pH 7.4, 0.1% w/v Tween 80) was prepared to mimic the physiological pH conditions. An accurately weighed aliquot of GLNP dispersion or free GL solution equivalent to 5.0 mg GL was transferred into a prehydrated cellulose acetate dialysis membrane (molecular weight cutoff 12 kDa, Biotopped Life Sciences, Beijing, China), sealed, and immersed in 100 ml of pre-equilibrated release medium contained in amber glass vessels. Incubation was performed at 37 ± 0.5 °C with horizontal orbital shaking at 100 rpm (Thermo Scientific MaxQ 4000). Evaporation was prevented by parafilm covers. Sampling was conducted at predetermined intervals (0.5, 1, 2, 4, 8, 12, 24, 48, 72, 96, 120, 144, and 168 h). At each time point, 1 ml of receptor medium was withdrawn and replaced with an equal volume of fresh medium. Samples were centrifuged (13,000*g*, 10 min, 20 °C), filtered [0.22 μm polyvinylidene difluoride (PVDF)], and analyzed for GL by HPLC with UV detection. The values were adjusted for dilution at each sampling point, and the cumulative release percentage (%) was calculated.

### Fabrication of sMNs

sMNs were fabricated by a vacuum-assisted micro-molding technique [30], using 1 cm × 1 cm polydimethylsiloxane (PDMS) molds containing a 15 × 15 array of conical cavities (600 μm height, 300 μm base diameter, ~5 μm tip). A 2-layer architecture, drug-loaded tips, and a drug-free backing were employed to ensure reliable skin insertion and rapid dissolution. First, a 5% (w/v) sodium alginate hydrogel was prepared in sterile ultrapure water (Fig. S2); for active formulations, GLNPs, TS, Rh.B, or their combinations were dispersed or dissolved in the sodium alginate hydrogel. After degassing under reduced pressure for 10 min, 60 μl of the hydrogel was spread evenly onto each PDMS mold. The molds were placed in a vacuum oven (600 mmHg, 25 °C, 10 min) to penetrate the hydrogel into the micro-cavities and then centrifuged (2,500*g*, 30 min, in a swinging bucket) to compact the tips and expel residual bubbles. Excess hydrogel was carefully scraped off, and the vacuum–centrifugation cycle was repeated once. The filled molds were placed in an oven and dried at 38 °C for 9 h to allow for complete solvent evaporation and tip solidification. For the backing layer, a solution was prepared by dissolving 30% w/w PVP and 1.5% w/w glycerol in ultrapure water. The mixture was stirred thoroughly to obtain a homogeneous solution. Then, 1 ml of this backing layer solution was cast onto the mold surface containing dry microneedles and briefly centrifuged (1,000*g*, 5 min) to ensure uniform contact. The microneedle patch was then dried in a vacuum oven (under atmospheric pressure) at 38 °C for 12 h to form a smooth, bubble-free backing layer. The solidified microneedle patch was gently demolded, inspected for integrity, sealed in aluminum pouches with desiccant, and stored at 4 °C until use. TS and GLNP-loaded sMNs were prepared under light-free conditions. All procedures were conducted under aseptic conditions in a class II biosafety cabinet to minimize microbial contamination.

### Characterization of microneedle patches

#### Morphological analysis

Macroscopic inspection was performed with a digital camera for both blank and Rh.B-loaded microneedle patches (sMN and Rh.B@sMN). SEM was then used to quantify tip sharpness, length, and surface texture.

#### Mechanical strength evaluation

A uniaxial compression assay (at 25 °C and 50 ± 2% RH) was carried out on a texture analyzer (TA.XTC-20, Bosin, Shanghai) fitted with a 2-mm-diameter stainless-steel probe. Microneedle arrays were fixed on the platform with tips oriented upward and centered under the probe. The probe descended at 0.01 mm/s until complete failure, and force–displacement curves were recorded. The failure force (F_max) was defined as the first inflection point on the stress–strain curve, and the force per needle was calculated as F_max divided by the number of needles in the compressed area.

#### Drug distribution and dissolution kinetics

The distribution of Rh.B dye (model drug) within the microneedles was visualized by laser confocal microscopy (LCM) (Nikon ECLIPSE Ti, 555 nm excitation, 580 to 620 nm emission). Stacks were acquired at 35-μm intervals along the *z* axis and reconstructed into 3-dimensional (3D) maps with NIS-Elements Viewer. The fluorescence throughout the microneedle shaft was measured. Furthermore, a 4% (w/v) agar gel (Biowest, France) maintained at 32 °C was selected as a surrogate matrix because its storage modulus (≈ 90 to 110 kPa at 1 Hz) lies within the mid-range reported for human HS tissue (50 to 150 kPa). Arrays were inserted to full depth to subject the entire needle shaft to compressive and shear stresses comparable to those encountered in vivo. Microneedle patches were retrieved after different time intervals and immediately imaged under an Olympus SZ61 stereomicroscope. Complete dissolution was defined as the time point at which ≤5% of the original height remained. The same procedure was employed for in vivo dissolution studies using a rabbit ear HS model.

### Ex vivo permeation studies of TS+GLNPs@sMN

Franz diffusion cells (effective area 1.0 cm^2^, receptor volume 12 ml) mounted with either full-thickness normal or HS rabbit skin (~1 mm thickness, harvested from rabbit ears 5 weeks post-scar induction) served as the permeation barrier. The dermal side was bathed with pH 7.4 PBS containing 4% (w/v) bovine serum albumin (BSA) and 0.1% (v/v) Tween 80 to simulate physiological protein-binding conditions. The receptor phase was maintained at 37 ± 0.5 °C and magnetically stirred at 600 rpm. Before mounting, the stratum corneum surface was gently swabbed with 70% ethanol and allowed to air-dry. A single TS+GLNPs@sMN patch was applied perpendicular to the skin surface with consistent manual pressure for 10 to 15 s. Receptor samples (0.5 ml) were withdrawn at predetermined times and immediately replaced with an equal volume of fresh, pre-equilibrated medium. Samples were deproteinized by mixing with acetonitrile (1:3 v/v) to precipitate BSA prior to HPLC analysis and then centrifuged (13,000*g*, 10 min, 20 °C), and the supernatant was filtered (0.22 μm PVDF) for quantification of total GL and TS by HPLC-UV. Sink conditions were maintained throughout the experiment. The cumulative permeated drug amount was corrected for dilution and normalized to cm^2^. Lag time, steady-state flux (Jss), and permeability coefficient (Kp) were calculated from the linear portion of the curve.

### In vivo anti-fibrotic study in the HS model

Female New Zealand White rabbits (*n* = 32) were used to assess in vivo anti-fibrotic efficacy. On the day of surgery, the ventral surface of each ear was shaved and disinfected with 0.5% (w/v) povidone iodine. Anesthesia was induced by intravenous injection of 1% pentobarbital sodium (3 ml kg^−1^) via the marginal ear vein. Three full-thickness wounds (diameter 5 mm, depth to cartilage) were created on the ventral surface of each ear using a sterile biopsy punch, spaced around 1 cm apart to avoid wound-edge interference. Hemostasis was achieved with gentle pressure using sterile gauze; wounds were then rinsed with sterile 0.9% NaCl, swabbed with 0.5% povidone iodine, and covered with an occlusive polyurethane film dressing (Tegaderm, 3M). Dressings were changed every 48 h under brief isoflurane anesthesia. Seven days post-injury, rabbits were re-anesthetized, and adherent scabs were gently removed with saline-soaked gauze to ensure uniform epithelialization. By day 30, all wounds had progressed to well-defined, erythematous, elevated scars characteristic of HS, as confirmed by visual inspection and vernier caliper measurement (height averaging 3 mm). Rabbits were randomly assigned to 8 groups (*n* = 4): normal (naive, no surgery, no treatment), control (HS induction without treatment), TS (intralesional treatment with TS), GLNPs (intralesional treatment with GLNPs), sMN, TS@sMN, GLNPs@sMN, and TS+GLNPs@sMN. Treatments were administered weekly, with microneedles applied until complete dissolution and intralesional injections delivered using a 30-gauge needle inserted tangentially into the scar tissue. All treatments were administered on days 1, 7, 14, and 21. Photographs of the scars were taken on days 0, 14, and 28. Throughout therapy, scar thickness was measured with a digital caliper to monitor treatment efficacy. All groups were visually monitored during behavioral studies to observe scratching at the scar area, assess itching, and evaluate the potential effects of therapy. Furthermore, the scar healing rate was calculated using the following formula:

$$HS\;\text{healing rate}\;\left(\%\right)=\left(1-\frac{\text{Scar area}\;at\;\text{time point}}{\text{Initial scar area}}\right)\times 100\%$$ (4)

Seven days after the final dose (day 28), rabbits were euthanized via intracardiac injection of pentobarbital under deep anesthesia. Subsequently, full-thickness scar tissue samples were collected. One set of tissue samples was designated for histopathological and immunohistochemical analyses. Another set of tissue was snap-frozen in liquid nitrogen and stored at −80 °C for subsequent biochemical assay.

### Statistical analysis

To ensure the reliability and reproducibility of the results, all experiments were performed in triplicate. Data are represented as the mean ± standard deviation (SD). For statistical analysis, the entire dataset was evaluated using GraphPad Prism (version 8.0.2) and ImageJ (version 1.52a). Comparisons between the 2 groups were conducted using an unpaired *t* test. Statistical significance was determined as follows: *P < 0.05, **P < 0.01, ***P < 0.001, and ****P < 0.0001.

## Results

### Preparation and characterization of GLNPs

**Fig. 2.**
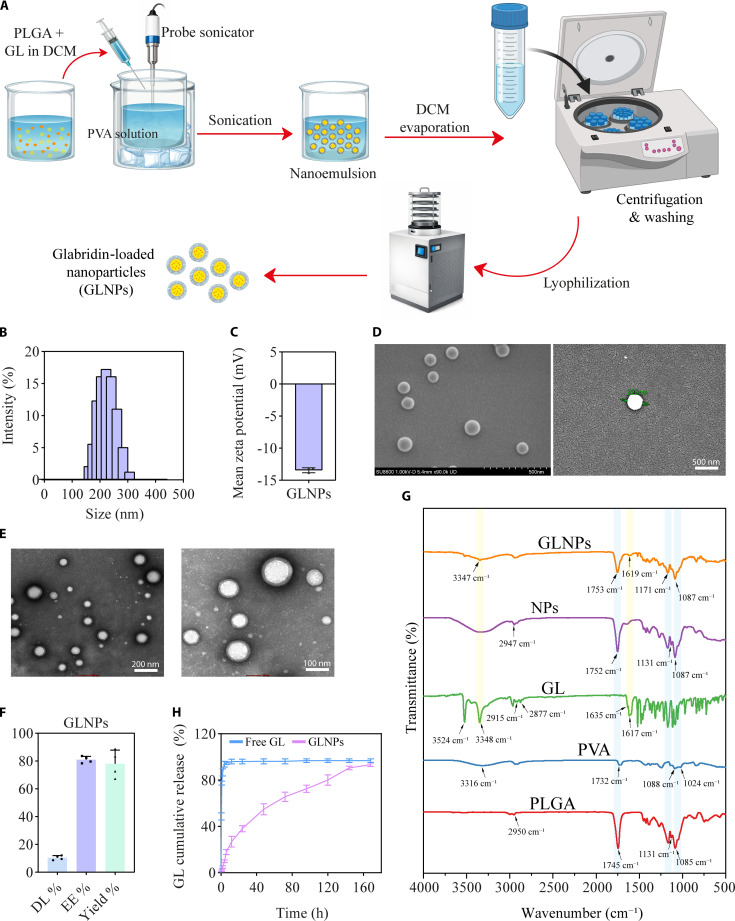
Preparation and characterization of glabridin-loaded nanoparticles (GLNPs). (A) Schematic illustration of GLNP preparation workflow. (B) GLNP particle size and (C) zeta potential (*n* = 3, mean ± SD). (D) SEM (scale bar, 500 nm) and (E) TEM images depicting GLNP morphology (scale bars, 200 and 100 nm). (F) GLNP drug loading (DL %), encapsulation efficiency (EE %), and yield (%) (*n* = 4, mean ± SD). (G) FTIR spectra of PLGA, PVA, GL, NPs, and GLNPs with characteristic peaks. (H) Cumulative GL release from GLNPs over 160 h (*n* = 3, mean ± SD).

The GLNPs were successfully synthesized via a modified single emulsion method, as depicted in Fig. 2A. Subsequent comprehensive characterization of the GLNPs’ physicochemical properties revealed a hydrodynamic diameter of 234.25 ± 5.93 nm, which is indicative of a relatively uniform size distribution (Fig. 2B). The low PDI of 0.18 ± 0.02 further supports the high uniformity and narrow size distribution of the GLNPs. The zeta potential of the GLNPs, measured at −13.43 ± 0.31 mV (Fig. 2C), suggests a stable colloidal dispersion, attributed to the repulsive forces arising from the negative charge on the particle surfaces. Morphological analysis via SEM and TEM (Fig. 2D and E) confirmed that the GLNPs were spherical with a smooth surface, which is indicative of successful encapsulation of GL within the PLGA matrix. The absence of agglomeration or surface irregularities suggests a high degree of uniformity in the GLNP preparation process. The DL % of the GLNPs was quantified at 10.41 ± 0.87%, while the EE % was 81 ± 1.3%. The overall yield of the GLNP formulation was 78 ± 5.4% (Fig. 2F), demonstrating the efficiency of the employed method of synthesis. The FTIR analysis (Fig. 2G) provided insights into the chemical composition and interactions within the synthesized GLNPs. The FTIR spectrum of PLGA showed characteristic peaks at 1,745, 1,085, and 1,131 cm^−1^, corresponding to the carbonyl (C=O) stretching vibrations of ester groups, and a broad peak at 2,950 cm^−1^ due to C–H stretching vibrations of methyl and methylene groups. GL exhibited a peak at 1,635 cm^−1^ for C=O stretching, with broad peaks at 3,348 and 3,524 cm^−1^ indicating O–H stretching vibrations of phenolic groups [31], and peaks at 2,877 and 2,915 cm^−1^ for C–H stretching. PVA’s spectrum featured a strong peak at 1,732 cm^−1^ for C=O stretching, with peaks at 1,024 and 1,088 cm^−1^ for C–O–C stretching and a broad peak at 3,316 cm^−1^ for O–H stretching. The NP spectrum displayed peaks at 1,752, 1,087, and 1,131 cm^−1^, similar to PLGA, and a peak at 2,947 cm^−1^ for C–H stretching. The GLNP spectrum integrated characteristic peaks from both PLGA and GL, confirming the successful encapsulation of GL. The peak at 1,753 cm^−1^ corresponded to PLGA’s C=O stretching, while peaks at 1,087 cm^−1^ and 1,171 cm^−1^ were associated with ester carbonyl groups. The peak at 1,619 cm^−1^ indicated GL’s C=O stretching, and the broad peak at 3,347 cm^−1^ was due to O–H stretching vibrations. These findings validate the successful incorporation of GL into the PLGA matrix of the NPs. Moreover, in vitro release studies revealed distinct kinetics between free GL and GLNPs. The free GL solution rapidly released over 90% of its content within the first 1.5 h, as shown by the sharp increase in cumulative release (Fig. 2H). In contrast, GLNPs displayed a controlled, biphasic release profile. During the initial 24 h, the GLNPs exhibited a rapid release of 38 ± 2.16% of the encapsulated GL. This phase is characterized by the release of drug molecules located on the surface of the NPs or within the outer layers of the PLGA matrix, which are more readily accessible for immediate release. The rapid release within this timeframe suggests that a notable portion of the drug is available for therapeutic action shortly after administration, potentially providing an initial therapeutic effect. Following the initial 24-h rapid release, the drug release rate decreased considerably, entering a sustained release phase that continued over the subsequent 144 h (from day 1 to day 6). During this phase, the cumulative release of GL increased gradually, reaching 93.33 ± 1.25% by the end of the study period (168 h). The sustained release is attributed to the controlled degradation of the PLGA polymer and the diffusion of the remaining drug through the polymer matrix. This extended-release profile suggests that NPs can sustain therapeutic drug levels, potentially improving efficacy and reducing dosing frequency.

### Preparation and characterization of microneedle patches

**Fig. 3.**
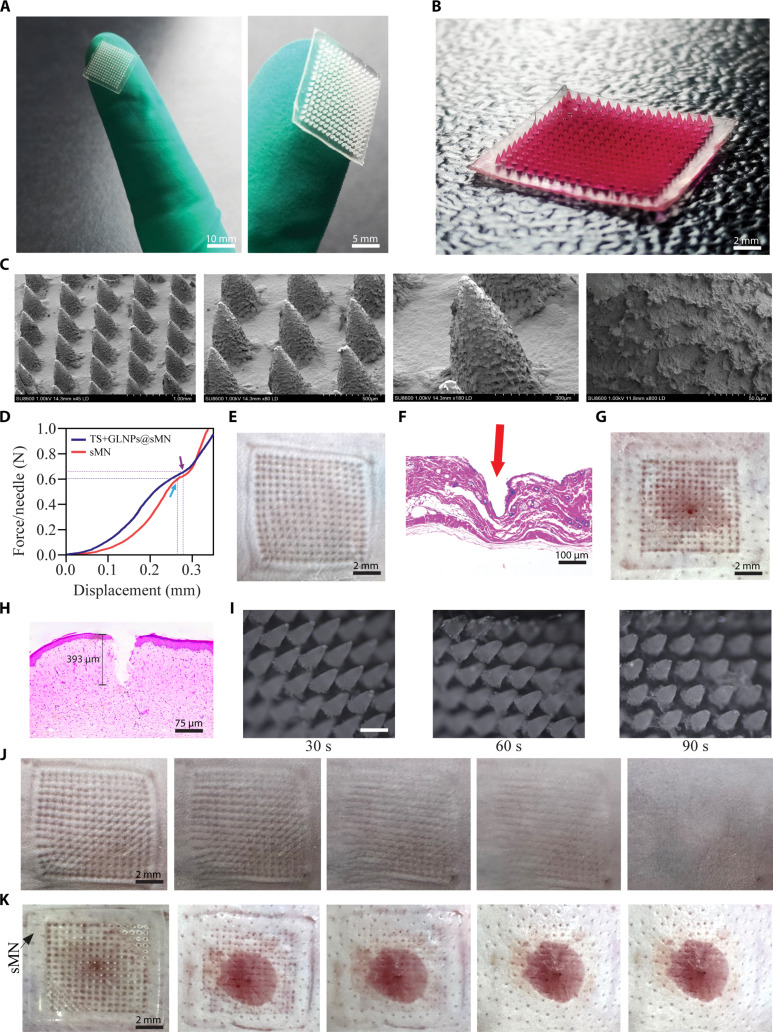
Characterization of sodium alginate-based microneedle patch (sMN). (A) Photograph of sMN patch on a finger for size reference (scale bars, 10 and 5 mm). (B) sMN patch loaded with Rh.B for visualizing drug distribution (scale bar, 2 mm; magnification, 2×). (C) SEM images depicting sMN morphology at various magnifications. (D) Mechanical strength comparison between sMN and TS+GLNPs@sMN. (E) Micro-pinholes in mouse skin post-insertion of sMN patch and (F) H&E-stained histological section displaying the microneedle-induced hole (indicated by red arrow) (scale bar, 100 μm). (G) Micro-pinholes in rabbit ear HS and (H) H&E-stained section showing microneedle-induced penetration depth (scale bar, 75 μm). (I) Microneedle integrity evaluation in HS model at 0, 60, and 90 s post-insertion (scale bar, 500 μm). (J) Time-lapse of skin recovery in mice following sMN insertion (scale bar, 2 mm; magnification, 2×). (K) Skin recovery in the rabbit ear HS over time following sMN application (scale bar, 2 mm; magnification, 2×).

Microneedle arrays were successfully engineered using a 5% (w/v) sodium alginate hydrogel. Digital photography of the resulting sMN and Rh.B@sMN patches demonstrated uniform, symmetric conical microprojections integrated into a PVP backing layer (Fig. 3A and B). The backing layer was observed to be homogeneous, devoid of bubbles, and exhibited mechanical pliability. Subsequent SEM analysis (Fig. 3C) confirmed the conical geometry of the sMN, with an average height of 565 ± 12 μm. Furthermore, the axial compression testing of the sMN demonstrated a linear stress–strain relationship up to the failure point, as shown in Fig. 3D. The sMN exhibited an average bending force of 0.61 ± 0.03 N per tip. In comparison, TS+GLNPs@sMN displayed a slightly higher bending force of 0.65 ± 0.04 N per tip, due to successful loading of TS and GLNPs. Despite exhibiting bending deformation rather than fracture, the microneedles (sMN and TS+GLNPs@sMN) possessed a compressive strength that exceeded the ~0.5 N per tip threshold previously reported as sufficient for HS penetration [2,24], establishing their mechanical robustness for insertion. Furthermore, the backing layer, which is critical for handling, conformability, and user comfort, was assessed for thickness and weight. The sMN had a mean weight of 5.6 ± 0.4 mg, while the backing layer weighed 10.09 ± 0.61 mg (Table S1) and measured 0.75 ± 0.12 mm in thickness. A linear relationship was observed between the volume of the casting solution and both the mass and thickness of the backing layer. The total sMN patch mass, approximately 15.7 mg, was markedly lower than commercial transdermal systems, ensuring negligible mechanical load and maximal patient acceptability. Application of the sMN patch produced well-defined, uniformly spaced micropores on both the mouse dorsal skin and the rabbit HS tissue (Fig. 3E to G). Histological analysis (Fig. 3F to H) immediately after patch removal (*t* ≈ 5 s) demonstrated micro-channel depths of 523 ± 8 μm in mouse skin and 392 ± 5 μm in rabbit ear HS tissue, successfully traversing the hyperplastic epidermis to reach the upper dermis. For microneedle integrity evaluation, patches were inserted into HS and retrieved at 0, 60, and 90 s. As shown in Fig. 3I, no evidence of needle fracture was observed; instead, the microneedles exhibited tip bending, softening, and progressive dissolution characteristic of fast-dissolving alginate matrices. Furthermore, skin insertion and recovery tests depicted in Fig. 3J and K demonstrate the rapid healing dynamics of the microchannels post-sMN removal. Microchannels closed within 2 h post-removal, confirming rapid skin barrier recovery and minimal invasiveness.

#### Drug distribution and dissolution studies of sMN

**Fig. 4.**
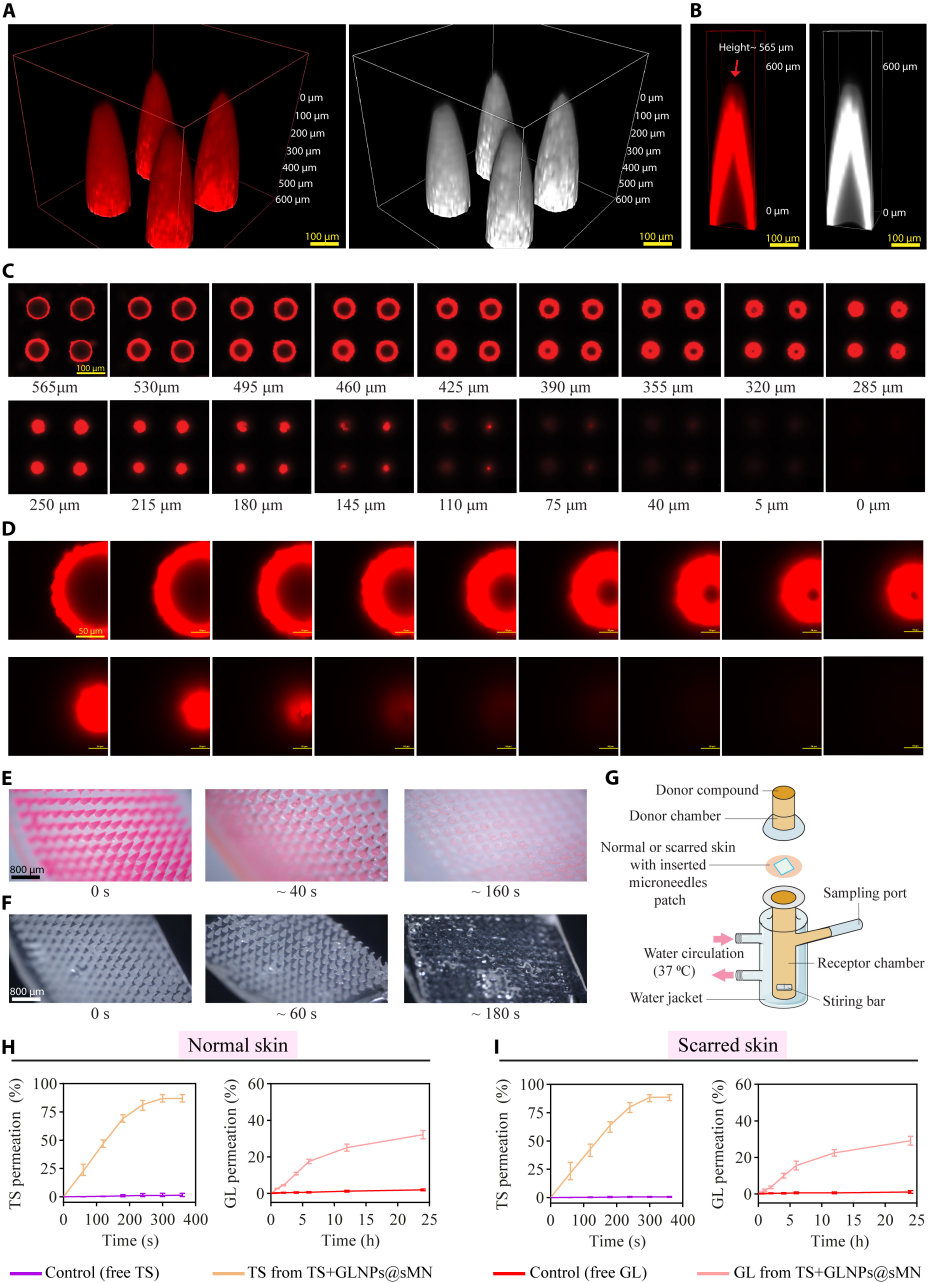
Evaluation of microneedle drug distribution, dissolution, and release kinetics. (A) Images of 3D LCM reconstruction of Rh.B-loaded microneedles (Rh.B@sMN) delineating the microneedle morphology and internal drug distribution, shown with a corresponding grayscale image (scale bar, 100 μm). (B) Cross-sections of a single Rh.B@sMN tip displaying Rh.B distribution, with a corresponding grayscale image (scale bar, 100 μm). (C) Sequential tomographic slices from LCM scans of Rh.B@sMN, showcasing drug presence from the base to the tip (scale bar, 100 μm). (D) Sequential cross-sectional slices of a single Rh.B@sMN tip (scale bar, 50 μm). (E) In vitro dissolution of Rh.B@sMN observed under optical microscopy (scale bar, 800 μm). (F) In vivo dissolution of sMNs in HS model (scale bar, 800 μm). (G) Franz diffusion cell setup for ex vivo permeability studies. (H) Ex vivo permeation profiles of TS and GL across rabbit normal ear skin, quantified over time (*n* = 6, mean ± SD). (I) Ex vivo permeation profiles of TS and GL across HS, quantified over time (*n* = 6, mean ± SD).

The spatial distribution of Rh.B within the microneedles was characterized using 3D reconstruction by LCM. The analysis revealed a drug concentration that was highest at the periphery and tip of the microneedles, extending uniformly along the entire length of the 565-μm projections (Fig. 4A). The cross-sectional imaging (Fig. 4B to D) also demonstrated a consistent Rh.B fluorescence pattern that traced the microneedle contours, with fluorescence intensity peaking at the microneedle tips. The bottom-up tomographic analysis (Fig. 4C and D) illustrated a gradual reduction in the diameter of the projected circles from the base to the tip, confirming the conical geometry and homogeneous axial drug loading of the microneedles. Furthermore, to assess the dissolution behavior, sMNs were evaluated in both a controlled surrogate model and scar tissue. In 4% agar gel at 32 °C (Fig. 4E), a standard homogeneous matrix for microneedle assessment, complete dissolution occurred within 160 ± 20 s. In contrast, in vivo dissolution in rabbit ear HS tissue required 180 ± 20 s for complete dissolution (Fig. 4F), due to the denser collagen matrix and reduced water activity characteristic of scar tissue. The complete microneedle dissolution occurred without fracture, characterized by progressive tip deformation, softening, and eventual disintegration (Fig. S3). The rapid sMN dissolution enables immediate TS release into microchannels, while GLNPs retained in the epidermis provide sustained GL release, a dual mechanism enhancing HS therapy.

#### GLNP size in sMN, loading capacity, and ex vivo permeation in TS+GLNPs@sMN

Upon integration into the sMN matrix, GLNPs exhibited stable physicochemical properties, with a hydrodynamic diameter of 252 ± 4.3 nm, a PDI of 0.21 ± 0.03, and a zeta potential of −14.43 ± 0.46 mV (Fig. S4A and B and Table S1). The SEM image confirms spherical morphology with smooth surfaces and absence of particle fusion or PLGA annealing (Fig. S4C). Moreover, TEM reveals an intact core–shell architecture (Fig. S4E). These data demonstrate that our mild fabrication conditions (38 °C, below PLGA glass transition temperature) preserve GLNP structural integrity without fusion or thermal degradation, ensuring sustained-release functionality throughout microneedle fabrication. Furthermore, the sMN array demonstrated a maximal drug loading capacity of 0.023 ± 0.004 mg for GL encapsulated within GLNPs and 0.031 ± 0.002 mg for TS (Table S1). The loading profiles of GLNPs and TS showed a competitive interaction, with an increase in loading of one compound resulting in a proportional decrease in the other. This competitive loading behavior suggests that the sMN matrix has a finite capacity for drug incorporation, and the presence of one drug can influence the loading efficiency of the other. Ex vivo permeation studies using a Franz diffusion cell (Fig. 4G) confirmed that TS+GLNPs@sMN exhibited the desired release kinetics. In normal rabbit skin, the permeation of TS from a control (free TS) solution was 1.25 ± 1.32% within 360 s. In contrast, TS permeation from TS+GLNPs@sMN was significantly higher, reaching 86.9 ± 2.35% (Fig. 4H and I). For GL, the permeation from a control solution was 1.94 ± 0.54% within 24 h, compared to 32.13 ± 2.31% from TS+GLNPs@sMN (*P* < 0.01). In HS, the permeation of free TS from a control solution was 0.57 ± 0.38% within 360 s, whereas TS permeation from TS+GLNPs@sMN was 88.81 ± 2.08% (*P* < 0.001). For GL, the permeation from a control solution was 1.08 ± 0.76% within 24 h, compared to 29.07 ± 2.49% from TS+GLNPs@sMN (*P* < 0.01) (Fig. 4H and I). Overall, the TS+GLNPs@sMN achieved rapid TS permeation and sustained GL release in both normal and scar tissue, with modestly reduced kinetics in HS attributable to its denser collagen matrix.

### In vitro anti-proliferative efficacy

**Fig. 5.**
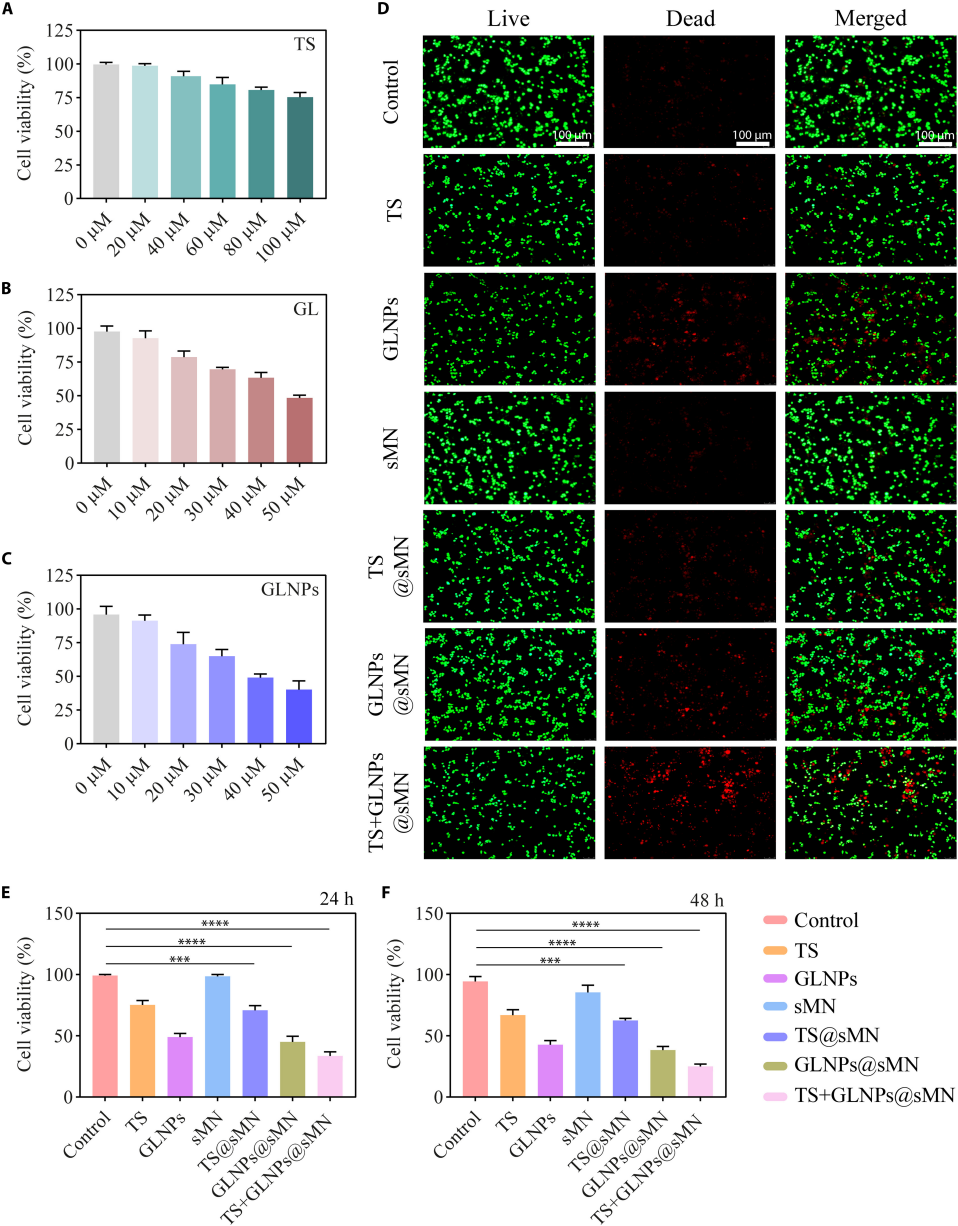
Cell viability of fibroblastic cells (HSFs) after 24 h of treatment with (A) TS, (B) GL, and (C) GLNPs at different drug concentrations. (D) Live-dead staining of cells treated with different formulations after 24 h (scale bar, 100 μm). (E) Cell viability of different formulations after 24 h and (F) 48 h (*n* = 3, mean ± SD, *P < 0.05, **P < 0.01, ***P < 0.001, and ****P < 0.0001).

The anti-proliferative effects of GL, TS, and GLNPs on HSFs were evaluated using a dose-dependent approach. The results, depicted in Fig. 5A to C, demonstrate a significant reduction in cell viability with increasing concentrations of each treatment. At 100 μM, TS reduced cell viability to 75.33 ± 2.87% (Fig. 5A). This indicates that TS exhibits dose-dependent anti-proliferative effects. Similarly, increasing concentrations of GL led to a dose-dependent decrease in cell viability (Fig. 5B). At 24 h, GL treatment reduced cell viability to 63.37 ± 3.25% at 40 μM and to 48.33 ± 1.70% at 50 μM. GLNPs exhibited a comparable trend, with more pronounced inhibition than GL alone. At 24 h, GLNPs reduced cell viability to 49.07 ± 2.29% at 40 μM and to 40.33 ± 5.25% at 50 μM (Fig. 5C). These results highlight the enhanced anti-proliferative efficacy of GLNPs compared to free GL, suggesting that NP formulation improves the potency of GL in inhibiting cell proliferation. Based on these findings, TS at 100 μM and GL at 40 μM were selected for further formulations. The cell viability at 24 h was substantially lower in all treatment groups compared to the control and sMN (Fig. 5E). The inhibitory effect of sMN was minimal due to the non-inhibitory nature of sodium alginate [32]. The TS@sMN formulation exhibited a cell viability of 71.0 ± 2.94%, while GLNPs@sMN showed a viability of 45.06 ± 3.70%, and the combination of TS+GLNPs@sMN resulted in a marked reduction in cell viability to 33.58 ± 2.70% at 24 h. After 24 h, the calcein-AM/PI double staining kit showed a similar trend to the CCK-8 assay, with the most pronounced reduction in cell viability observed for TS+GLNPs@sMN (Fig. 5D). After 48 h of treatments (Fig. 5F), TS showed a viability of 67.0 ± 3.56%, GLNPs had a viability of 42.77 ± 2.74%, and the combination formulations, TS@sMN (62.67 ± 1.25%) and GLNPs@sMN (38.37 ± 2.50%), demonstrated notable inhibition. Most strikingly, the combination of TS+GLNPs@sMN achieved the most excellent anti-proliferative effect, with a cell viability of 25.0 ± 1.63% after 48 h. This enhanced inhibitory effect is likely due to the synergistic action of TS and GLNPs, which together demonstrate the most potent anti-proliferative activity.

### Cell migration, angiogenesis, and in vivo biocompatibility evaluation

**Fig. 6.**
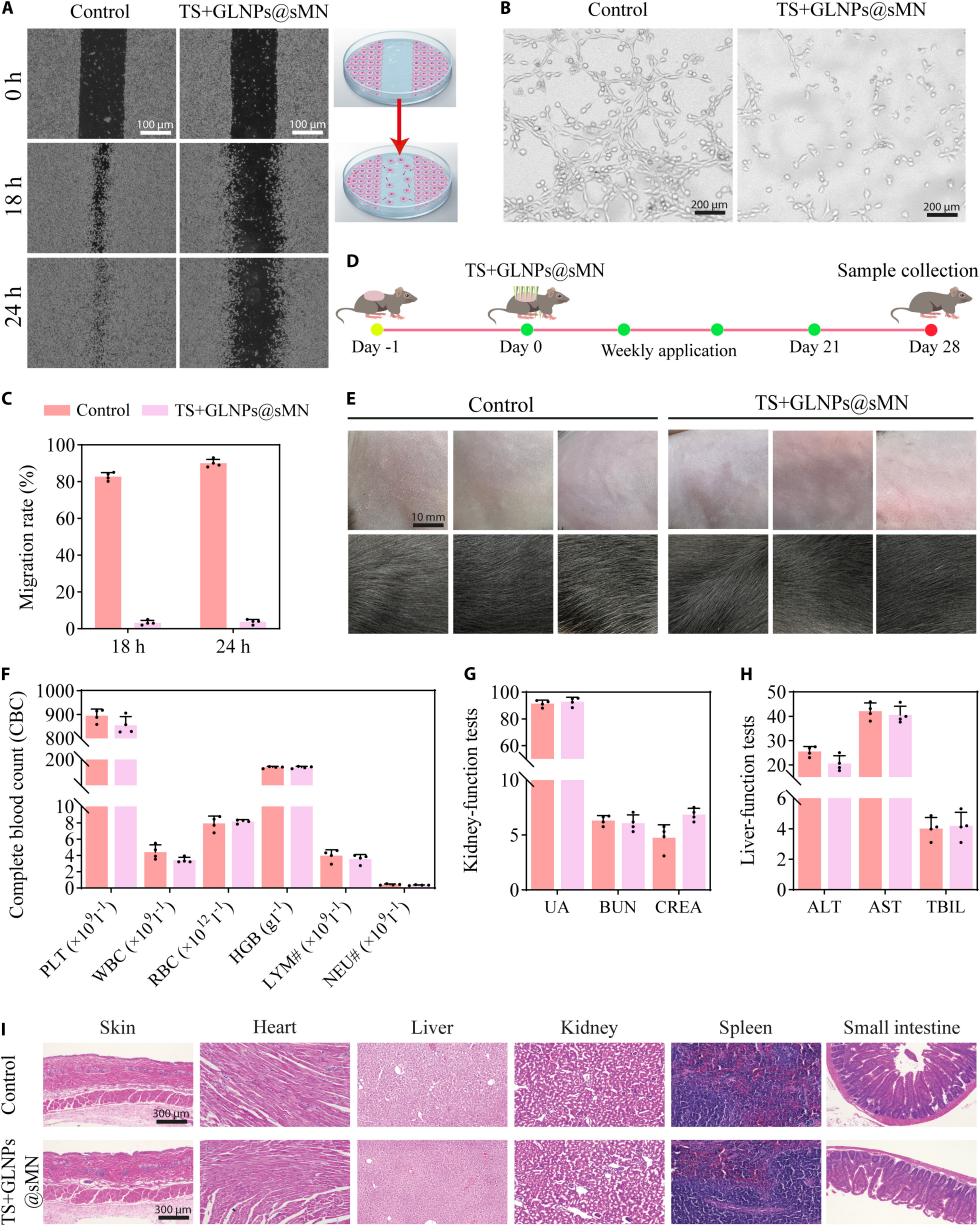
(A) Micrographs of HSF migration after 18 h and 24 h for control versus TS+GLNPs@sMN (scale bar, 100 μm). (B) Images of tube formation (scale bar, 200 μm). (C) Cell migration rate (%). (D) In vivo biocompatibility test timeline of TS+GLNPs@sMN patch. (E) Skin irritation test images (scale bar, 10 mm). (F) CBC parameters: platelet (PLT), white blood cells (WBC), red blood cells (RBC), hemoglobin (HGB), lymphocyte (LYM), and neutrophil (NEU) counts (*n* = 4, mean ± SD). (G) Serum renal function markers: uric acid (UA), blood urea nitrogen (BUN), and creatinine (CREA) (*n* = 4, mean ± SD). (H) Serum hepatic function markers: alanine aminotransferase (ALT), aspartate aminotransferase (AST), and total bilirubin (TBIL) (*n* = 4, mean ± SD). (I) H&E-stained sections of skin, heart, liver, kidney, spleen, and small intestine from control and TS+GLNPs@sMN-treated mice (scale bar, 300 μm) (*n* = 3).

A uniform monolayer of scratched HSF gap was tracked for 24 h. Control cultures re-epithelialized briskly, achieving 82.8 ± 1.9% closure at 18 h and 90.0 ± 1.9% by 24 h (Fig. 6A and C). TS+GLNPs@sMN reduced this response to 3.3 ± 1.1% and 3.8 ± 1.1%, respectively, corresponding to >96% inhibition at both intervals (*P* < 0.001 versus control). The near-complete arrest of fibroblast movement suggests that TS+GLNPs@sMN can limit the expansive migratory phase driving excessive ECM deposition in HS. Because fibroblast migration precedes and facilitates endothelial cell recruitment during scar vascularization, we next examined whether the suppressed motility phenotype extended to angiogenesis. After 4 h of exposure, control-treated HSFs assembled into highly organized, parallel arrays with numerous branch nodes and junctions, morphological hallmarks of active vascular plexus formation (Fig. 6B and Fig. S5). TS+GLNPs@sMN abolished this architecture; cells remained separated, with scarce branches and a 4-fold reduction in both node number and junction density (*P* < 0.0001). In the in vivo biocompatibility study, mice received the same topical dose used in the subsequent efficacy study and were monitored for 28 d (Fig. 6D). No mortality, behavioral abnormalities, or macroscopic cutaneous irritation were recorded, and the administration site retained normal architecture (Fig. 6E). Body-mass trajectories overlapped with those of the vehicle-treated animals throughout the observation period (Fig. S6). The complete blood count (CBC), including platelet (PLT), white blood cell (WBC), red blood cell (RBC), hemoglobin (HGB), lymphocyte (LYM), and neutrophil (NEU), remained within reference ranges (Fig. 6F), as did serum markers of renal function [uric acid (UA), blood urea nitrogen (BUN), creatinine (CREA)] and hepatic function [alanine aminotransferase (ALT), aspartate aminotransferase (AST), total bilirubin (TBIL)] (Fig. 6G and H). Histopathology of the TS+GLNPs@sMN-exposed skin, heart, liver, kidney, spleen, and small intestine revealed no inflammatory infiltrates, necrosis, or fibrosis (Fig. 6I). Collectively, these data demonstrate that TS+GLNPs@sMN is well tolerated at therapeutically relevant exposures, supporting its potential for long-term scar modulation in subsequent studies.

### In vivo anti-fibrotic efficacy in the HS model

**Fig. 7.**
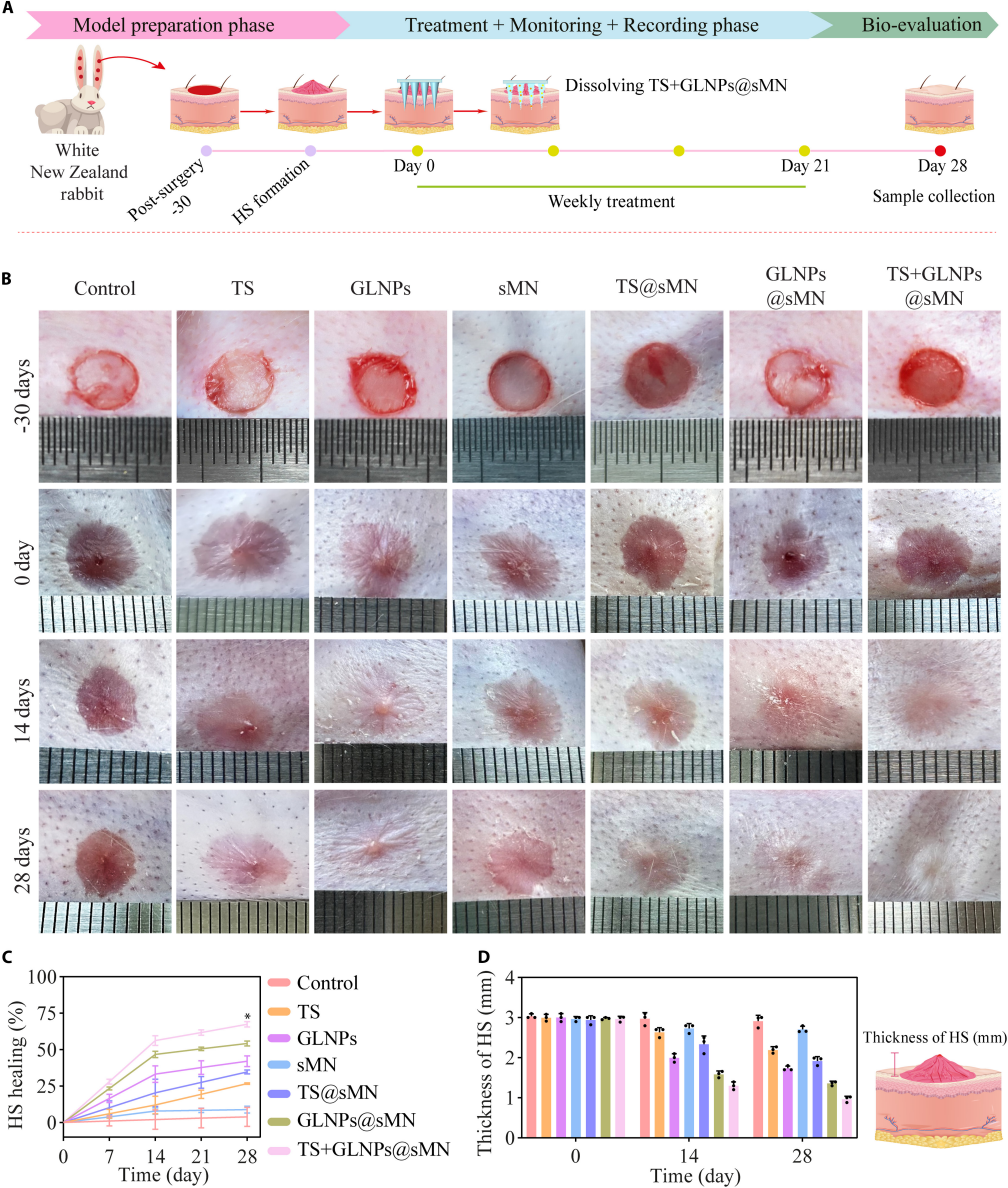
(A) Schematic flow of HS model induction, weekly microneedle application, and bio-evaluation sampling. (B) Images of rabbit ear from wound to successful HS formation and treatment progression. (C) HS healing rate (%) from day 0 to day 28 (*n* = 3, mean ± SD, *P < 0.05). (D) Thickness of HS (mm) on days 0, 14, and 28 (*n* = 3, mean ± SD).

The anti-fibrotic efficacy of TS+GLNPs@sMN was evaluated using an HS model established in rabbit ears. The experimental timeline, as depicted in Fig. 7A, outlined the model preparation, treatment administration, and subsequent monitoring phases. Prior to the initiation of treatments, the HS tissues were characterized by a reddish pink coloration and increased prominence, with an average thickness of approximately 3 mm, and a firmer texture compared to the surrounding normal skin (Fig. 7B). A 4-week treatment regimen was implemented, with weekly applications on days 0, 7, 14, and 21. During monitoring, groups treated with TS+GLNPs@sMN exhibited the least scratching , indicating that the formulation was more effective at mitigating itch. Furthermore, weekly monitoring of scar healing rate showed that the GLNPs@sMN and TS@sMN groups exhibited significantly superior healing compared with direct injection of GLNPs and TS into the HS bed. This suggests that incorporating these treatments into sMN enhances their therapeutic efficacy (Fig. 7C). Notably, the highest healing rate was observed in the TS+GLNPs@sMN treatment group. Photographic documentation of the treated HS was conducted on days 0, 14, and 28. A marked improvement in scar appearance was observed, particularly in the TS+GLNPs@sMN-treated group, which showed a coloration that closely resembled the adjacent tissues (Fig. 7B). This was also indicative of reduced inflammation and stabilization of mast cells. In contrast, the control group exhibited noticeable redness and swelling, reflecting mast cells in an unstable or overactive state that released pro-inflammatory mediators, contributing to the inflammation. Quantitative assessment of HS thickness was performed on days 0, 14, and 28 (Fig. 7D). Unlike control and blank sMN groups, active treatments significantly decreased HS thickness over 4 weeks, with TS+GLNPs@sMN outperforming both traditional intralesional injections and single-drug microneedles (TS@sMN and GLNPs@sMN). Post hoc power analysis of day 28 data confirmed the statistical robustness of these findings. The comparison between control (2.91 ± 0.15 mm) and TS+GLNPs@sMN (0.97 ± 0.08 mm) revealed a mean difference of 1.94 mm [95% confidence interval (CI): 1.68 to 2.21 mm] with an adequate size (Cohen’s *d* = 16.59) and observed power exceeding 99%, indicating that the sample size was sufficient to detect the pilot-scale observed effect.

### Histological and biochemical assay

**Fig. 8.**
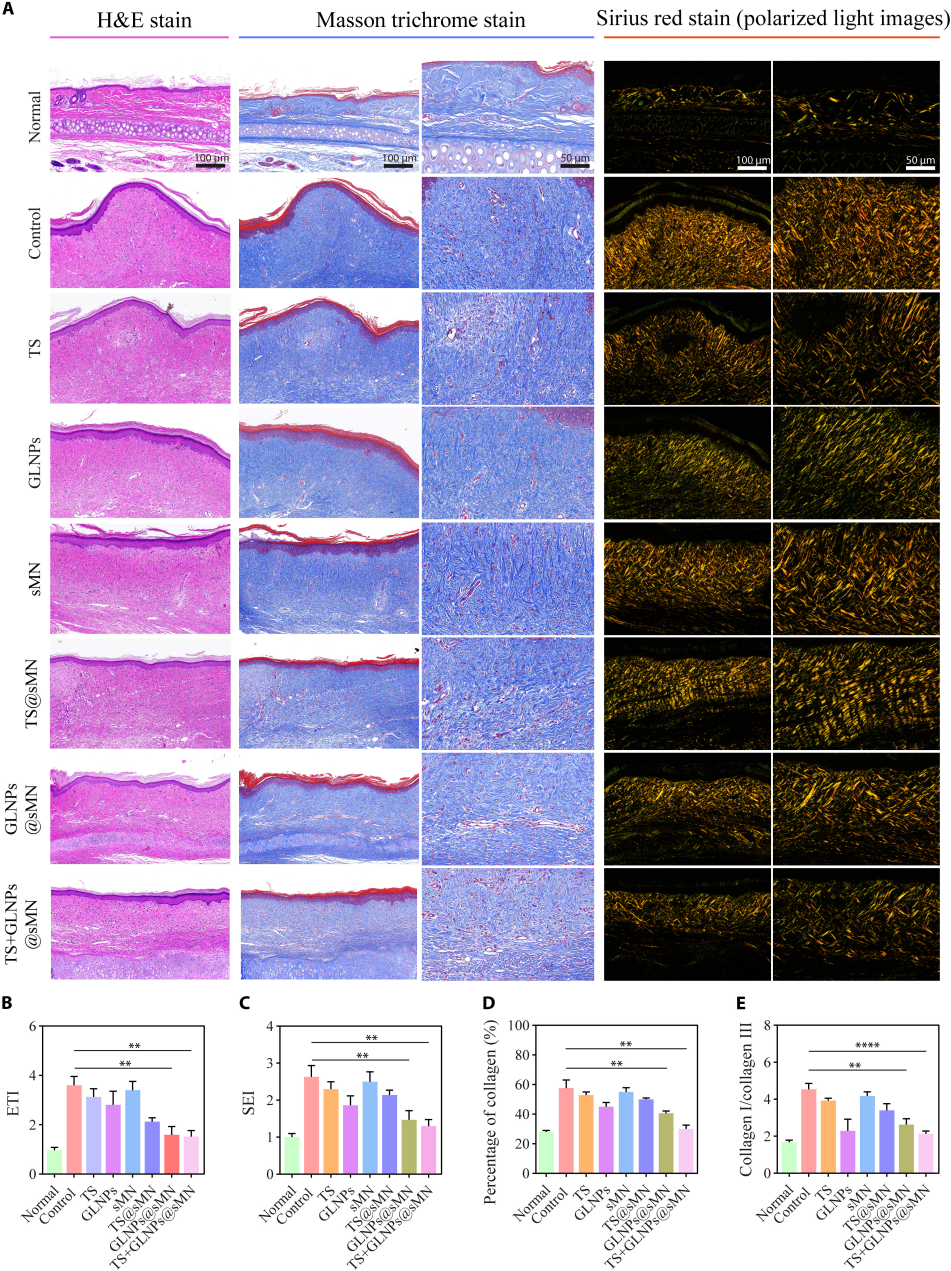
Comparative histological analysis of HS tissue across diverse treatment regimens. (A) Images of H&E (scale bar, 100 μm), Masson’s trichrome (scale bars, 100 and 50 μm), and Sirius red staining (scale bars, 100 and 50 μm). (B) ETI. (C) SEI. (D) Percentage of collagen (%) and (E) collagen I/collagen III ratio determined by Sirius red polarized light microscopy. ImageJ 1.52a, thresholding: collagen I (hue 0° to 40°), collagen III (hue 41° to 120°), 5 random fields per sample, *n* = 4, mean ± SD. *P < 0.05, **P < 0.01, ***P < 0.001, ****P < 0.0001.

**Fig. 9.**
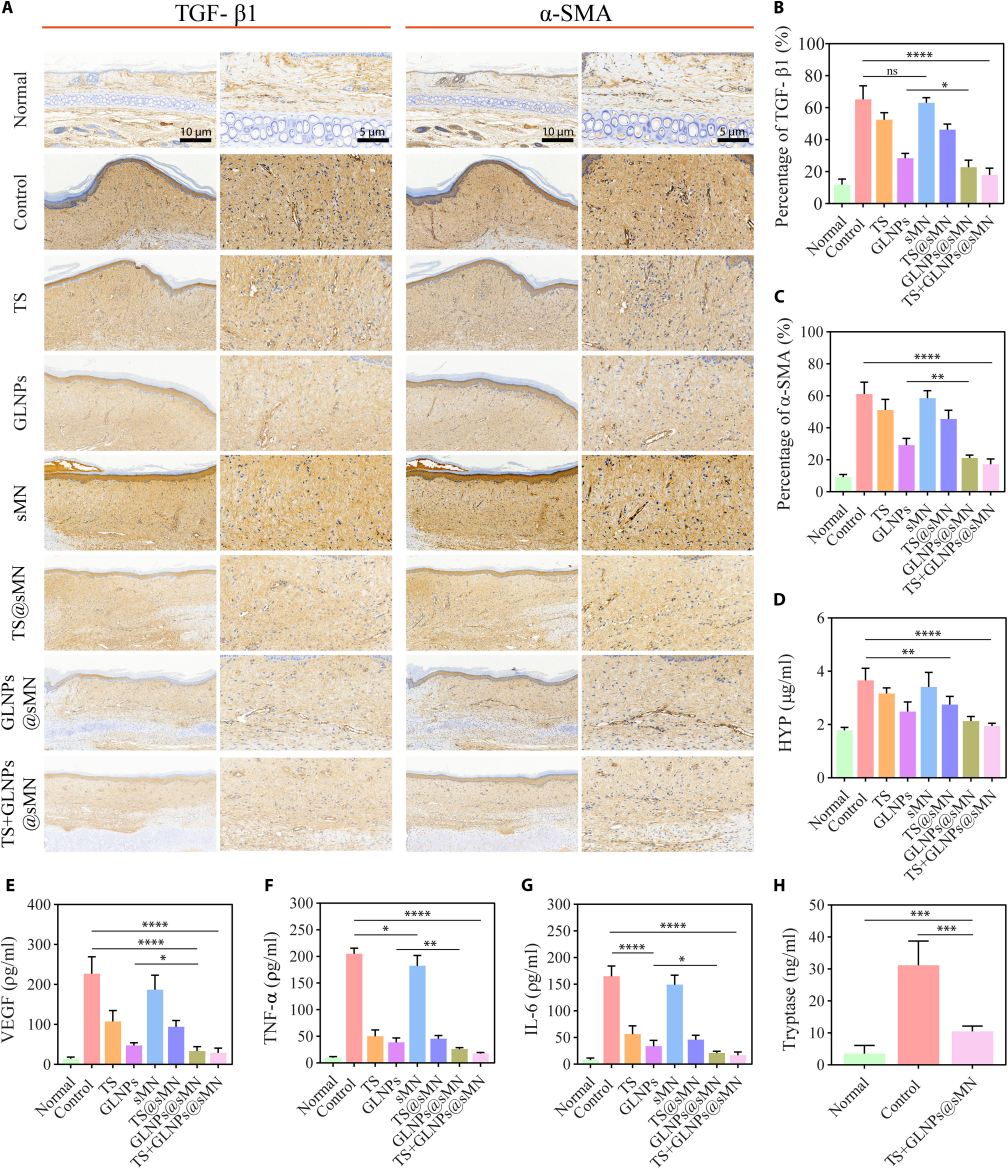
Comparative histological and biochemical assay analysis of HS tissue across diverse treatment regimens. (A) Immunohistochemical staining of TGF-β1 and α-SMA in HS tissue samples (scale bars, 100 and 50 μm). (B) Quantification of TGF-β1 and (C) α-SMA expression as a percentage of positive staining area. (D) HYP, (E) VEGF, (F) TNF-α, (G) IL-6, and (H) tryptase levels in tissue homogenates (*n* = 6, mean ± SD, *P < 0.05, **P < 0.01, ***P < 0.001, and ****P < 0.0001).

The histological analysis of HS models, using hematoxylin and eosin (H&E)-stained images, provided detailed insights into the efficacy of various treatments. The TS+GLNPs@sMN-treated group exhibited a histological structure closely resembling that of normal skin, characterized by a thin dermis and a much flatter epidermis (Fig. 8A). In contrast, other treatment groups displayed dermis hyperplasia or uneven dermis compared to the normal group. Previous studies have also established a positive correlation between increased epidermal thickness and scar severity [4]. Figure 8A illustrates a thick epidermis in the control group, while the TS+GLNPs@sMN group exhibited the thinnest and most even epidermis overall. The degree of epidermal hypertrophy was quantitatively assessed using the ETI, which was lowest in the TS+GLNPs@sMN group, indicating the least severe HS. The TS+GLNPs@sMN treatment was found to be more effective than traditional injections of TS and GLNPs, as well as TS or GLNPs in microneedles alone (Fig. 8B). The H&E-stained images also revealed the SEI, which measures the height of HS from ear cartilage to epidermis. As shown in Fig. 8C, the TS+GLNPs@sMN group demonstrated a significant reduction in HS height compared with the control group, while sMN alone had minimal impact. Notably, TS+GLNPs@sMN achieved near-complete reduction in HS height (SEI ≈ 1), closely resembling normal skin and corroborating the morphological analysis. Overall, the TS+GLNPs@sMN group exhibited the most significant reductions in epidermal and dermal thickness, as well as hyperemia. Masson’s trichrome staining was employed to visualize collagen fibers (blue) and muscular fibers (red) in HS tissues. The control group displayed significant dermal hyperplasia, increased blood vessels, and densely packed, disordered collagen fibers, covering 57.67 ± 4.50% of the area with blue collagen (Fig. 8A and D). While TS and TS@sMN treatments reduced the percentage of blue collagen area to some extent, GLNPs and GLNPs@sMN showed reductions to 45.0 ± 2.45% and 40.67 ± 1.25%, respectively. This difference may be attributed to the direct injection of TS or its rapid release from fast-dissolving microneedles. In contrast, GLNPs exhibited a sustained drug release effect. The TS+GLNPs@sMN group achieved the greatest reduction, with 30.0 ± 2.16% blue collagen, closely matching the normal group’s 28.07 ± 0.74% (Fig. 8A and D). Additionally, the organization of collagen fibers in the TS+GLNPs@sMN group was markedly improved, with a more regular and parallel arrangement (Fig. 7A), indicating significant inhibition of excessive collagen deposition. Sirius red staining under polarized light further highlighted differences in collagen type (Fig. 8A and Figs. S7 and S8). The control and sMN groups exhibited yellowish orange to red-like thick fibers, indicative of high collagen I content. In contrast, the TS, GLNP, TS@sMN, GLNPs@sMN, and TS+GLNPs@sMN groups showed greenish yellow fibers, suggesting the presence of collagen III. Quantitative analysis of the collagen I/collagen III ratio revealed a significant reduction in the TS+GLNPs@sMN group, achieving a ratio closest to that of normal skin (Fig. 8E). This finding underscores the better efficacy of TS+GLNPs@sMN in remodeling collagen secretion. Furthermore, to assess fibrosis in treated HS models, we analyzed the expression of key fibrosis-related proteins using immunohistochemistry. TGF-β1 and α-SMA are critical profibrotic markers; TGF-β1 drives ECM accumulation, and α-SMA indicates myofibroblast activity. Our results showed that TS+GLNPs@sMN significantly suppressed TGF-β1 and α-SMA expression (Fig. 9A to C), leading to reduced ECM deposition and mitigated HS severity. This highlights the efficacy of TS+GLNPs@sMN in treating HS by reducing fibrosis. To evaluate the collagen content, HYP levels were measured in homogenates of sample tissues treated with different formulations (Fig. 9D). The control group showed elevated HYP levels compared to the normal group, reflecting increased collagen synthesis in HS. Treatment with TS, GLNPs, TS@sMN, GLNPs@sMN, and TS+GLNPs@sMN led to a decrease in HYP levels. The TS+GLNPs@sMN group exhibited the most substantial reduction, indicating effective collagen synthesis inhibition and potential for scar remodeling. The expression of TGF-β1 was significantly reduced in skin homogenate samples (Fig. S9), findings that were consistent with immunohistochemistry results. Notably, the most pronounced reduction in TGF-β1 was observed with TS+GLNPs@sMN treatment, indicating a robust anti-fibrotic effect in HS. Furthermore, the levels of the growth factor VEGF (angiogenic marker), inflammatory cytokines (TNF-α and IL-6), and the mast cell marker (tryptase) were measured in tissue homogenates to evaluate the in vivo anti-angiogenic, anti-inflammatory, and mast cell stabilizing effects of different treatments (Fig. 9E to H). The control group showed significantly higher levels of VEGF, TNF-α, IL-6, and tryptase than the normal group, indicating increased angiogenesis, inflammation, and mast cell activation in HS. Treatment with TS, GLNPs, TS@sMN, GLNPs@sMN, and TS+GLNPs@sMN resulted in a decrease in these markers. Notably, the TS+GLNPs@sMN group demonstrated the most significant reduction in VEGF, TNF-α, IL-6, and tryptase levels, suggesting a potent anti-HS effect. Tryptase promotes histamine release from mast cells, amplifying inflammation and contributing to itching and allergic symptoms. Therefore, a reduction in tryptase levels may indicate relief from itching.

## Discussion

HSs are a clinical challenge stemming from the abnormal healing of post-burns, surgery, trauma, or severe acne wounds. [33]. The etiology of HS is rooted in the disruptions of the wound-healing cascade, encompassing the stages of inflammation, proliferation, and remodeling [34,35]. Current treatments for HS suffer from substantial limitations, including high recurrence rates, invasive administration, and strong dependence on clinician expertise, all of which compromise patient compliance. Although TS has demonstrated anti-fibrotic and anti-pruritic effects [8] and GL has shown promise in inhibiting fibroblast proliferation and collagen deposition [17,18], their spatiotemporal coordination within a single self-administrable system with programmed sequential release kinetics has not been reported previously. This study successfully developed a novel, fast-dissolving sMN integrating TS and GLNPs (TS+GLNPs@sMN). Our design employs formulation-based kinetic control; the fast-dissolving alginate matrix ensures immediate TS release, while the PLGA NP architecture provides sustained GL release over 7 d. This time-programmed, reactive oxygen species (ROS)-independent approach ensures reliable drug delivery regardless of scar heterogeneity or disease stage. The selection of sodium alginate as the microneedle matrix was strategically used because of its unique physicochemical properties. As a natural, linear biocompatible polysaccharide, alginate readily forms physical gels without additional polymers or cross-linking agents. We employed a 5% pure sodium alginate hydrogel, which dissolves substantially faster than covalently cross-linked alternatives, conferring the rapid dissolution behavior essential for immediate drug release. This dissolution is further facilitated by the pH-responsive deprotonation of alginate in the neutral-to-alkaline wound environment, which generates electrostatic repulsion forces that rapidly disintegrate the gel matrix [36]. Mechanistically, the cone-shaped microneedles (~565 μm height) exhibit sufficient mechanical strength (>0.58 N per needle) to reliably penetrate the stratum corneum and HS tissue [2], yet dissolve completely within 180 ± 20 s. This rapid dissolution minimizes tissue irritation while facilitating the immediate release of hydrophilic TS into the dermal microchannels. Concurrently, the embedded GLNPs remain deposited within the viable epidermis, ensuring sustained GL release as the PLGA matrix degrades. The dual-release design produces synergistic therapeutic effects. TS rapidly addresses acute inflammation, pruritus, and fibroblast activation, while subsequently delivered GL drives sustained scar matrix modulation through anti-fibrotic, anti-angiogenic, and pro-apoptotic mechanisms. The therapeutic synergy between TS and GL operates through complementary molecular pathways. GL predominantly triggers fibroblast apoptosis via mitochondrial-dependent mechanisms [14], whereas TS primarily attenuates TGF-β signaling to suppress fibroblast activation and ECM production [10]. Furthermore, once HS has developed, continued angiogenesis typically proves ineffective and may exacerbate scarring. Our TS+GLNPs@sMN patch demonstrated potent anti-angiogenic effects, with tube formation assays revealing disrupted vascular network formation and ELISA analysis confirming >90% down-regulation of VEGF protein levels. This dual inhibition of fibroblast migration and VEGF-dependent angiogenesis positions TS+GLNPs@sMN as a rational, multipronged intervention for both established HS prevention and treatment.

In vivo biocompatibility studies in mice demonstrated satisfactory safety over 28 d, with no mortality, skin irritation, or abnormalities in hematological, hepatic, or renal parameters. This favorable profile stems from the dual-release design. TS is well tolerated systemically, while PLGA-encapsulated GL provides sustained local delivery within safe therapeutic windows. Efficacy studies revealed significant reductions in scar thickness, collagen deposition, and expression of profibrotic (TGF-β1, α-SMA), inflammatory (TNF-α, IL-6), angiogenic (VEGF), and mast cell (tryptase) markers, alongside improved collagen architecture and reduced epidermal thickness. Decreased scratching behavior confirmed mast cell stabilization and anti-pruritic effects. These findings validate TS+GLNPs@sMN as a biocompatible, dual-action platform that integrates anti-fibrotic, anti-inflammatory, anti-angiogenic, and anti-pruritic mechanisms via differential drug release kinetics.

Despite these promising outcomes, our research acknowledges certain limitations. The study’s scope does not extensively delve into the specific molecular pathways influenced by TS and GL. Although the “OFF-to-ON” paradigm provides a coherent framework for observed phenotypic transitions, it should be regarded as a conceptual design strategy rather than definitive molecular evidence. Direct validation of causal mechanisms (e.g., caspase-3/7 activation, mitochondrial permeabilization, and lineage tracing) was beyond the scope of this proof-of-concept study. Future mechanistic studies employing terminal deoxynucleotidyl transferase-mediated deoxyuridine triphosphate nick end labeling (TUNEL) assays, quantitative caspase measurements, and lineage-specific reporter models are therefore essential to establish causal linkages between the observed phenotypes and underlying signaling pathways. While macroscopic endpoints (scar thickness, healing rate) demonstrated robust effects, the study was underpowered for biochemical secondary outcomes (cytokines, HYP), which exhibited higher interindividual variability. Future confirmatory studies should employ larger cohorts (*n* ≥ 6 to 8) to ensure adequate statistical power for these endpoints and enable dose–response characterization. In vivo safety evaluation was limited to a short-term (28-d) preliminary assessment. Long-term local skin effects, cumulative toxicity with repeated dosing, foreign body responses to residual PLGA, immunogenicity (lymphocyte subsets, anti-alginate/PLGA antibodies), and delayed hypersensitivity or granuloma formation remain to be evaluated. Chronic studies (≥3 to 6 months) are therefore required to address these endpoints before clinical translation. Moreover, future work must include head-to-head comparisons with clinical standard-of-care therapies, particularly intralesional corticosteroids (e.g., triamcinolone acetonide) and silicone-based treatments, to establish comparative efficacy, recurrence rates, and patient compliance profiles, which are essential for real-world contextualization. Our current study has laid the necessary groundwork, and future research should investigate the potential of this microneedle patch for treating additional fibrotic conditions, opening new avenues for its broader application.

### Conclusion

To improve the HS treatment, this study presents a novel, biocompatible, single-dose fast-dissolving sMN integrated with TS and GLNPs (TS+GLNPs@sMN). The TS+GLNPs@sMN had high drug loading capacity, rapid dissolution, efficient skin penetration, and sequential drug release kinetics. This patch also ensures effective delivery of both TS and GL to the target tissue. In vitro and in vivo evaluations confirmed its anti-pruritic, anti-proliferative, anti-migratory, anti-angiogenic, and anti-fibrotic effects. The dual-action strategy, combining rapid TS release with sustained GL delivery, can effectively address the complex pathophysiology of HS. This minimally invasive, self-administrable, and user-friendly treatment approach can offer substantial advantages over traditional therapies, demonstrating superior efficacy and better patient compliance. In conclusion, the TS+GLNPs@sMN patch shows great promise as a next-generation treatment for HS, presenting a novel solution to improve patient outcomes and advance scar management.

## Data Availability

Data will be made available on request.
