# Supplementary material for: Fast-Dissolving Sodium Alginate-Based Microneedle Patch Integrating Tranilast and Glabridin-Loaded Nanoparticles for Dual-Action Hypertrophic Scar Therapy
Source: Biomater Res. 2026 Apr 13;30:0351. doi: 10.34133/bmr.0351 (PMC13074273; doi:10.34133/bmr.0351)
Supplement: Supplementary 1 — Materials and Methods Figs. S1 to S9 Table S1 [file bmr.0351.f1.docx]

**Supplementary Materials**

**Fast-Dissolving Sodium Alginate-based Microneedle Patch Integrating Tranilast and Glabridin-loaded Nanoparticles for Dual-Action Hypertrophic Scar Therapy**

Ayesha Younas^1,2,3^, Muhammad Sohail^4^, Yueting Li^1^, Quan Zhou^1^, Yonghui Shen^5^, Shuanghu Wang^1^, Yaru Shi^1^, Zhenqiu Shang^1*^, Chuxiao Shao^1*^, Jian Xiao^2,3*^

**Materials and methods**

**Weight and thickness of microneedle patch**

The weight of the microneedle tips was determined using a gravimetric method. Empty PDMS micromolds were first weighed (W_1_). The molds were then filled with sodium alginate hydrogel and dried in a vacuum oven at 38 °C for 9 h (under atmospheric pressure). The molds were re-weighed after drying (W_2_). The weight of the microneedle tips was calculated as the difference between the two weights (W_2_ – W_1_). Subsequently, a PVP backing layer was cast onto the dried microneedle tips. After the backing layer had dried (in a vacuum oven at 38 °C, 12 h, without vacuum), the assembly was weighed again (W_3_). The weight of the backing layer was determined by subtracting the weight of the microneedle tips (W_2_) from the total weight (W_3_), yielding the backing-layer weight (W_3_ – W_2_). The thickness of the backing layer was measured at five random positions using a calibrated digital Vernier caliper with a precision of ± 0.01 mm (n=5).

**Skin penetration and recovery studies**

One day before the experiment, the dorsal skin of anaesthetized mice and the scarred ear skin of rabbits were depilated and disinfected with 70 % (v/v) isopropanol. Microneedle patches were applied perpendicular to the surface and pressed with a thumb force of 4–5 N for 20 s. Micropore formation was immediately confirmed by the appearance of uniform perforations, which were photographed and documented with a digital macro lens camera. For histological evaluation, 8 mm full-thickness punch biopsies were collected immediately after microneedle patch removal and fixed in 4 % paraformaldehyde, paraffin-embedded, and sectioned at 5 µm. Sections were stained with hematoxylin–eosin (H&E) and examined under a light microscope to quantify stratum corneum disruption depth. Parallel macroscopic assessments tracked closure of the micro-channels over time in mouse skin and rabbit scarred skin. Digital images of the complete recovery were defined as the absence of visible perforations.

**Size of GLNPs after loading into microneedles and loading capacity**

To assess whether the casting and drying steps compromise the integrity of GLNPs, the microneedle tips from TS+GLNPs@sMN were carefully separated from the backing layer using a sharp scalpel. The tips were then dissolved in ultrapure water at 25 °C for 5 min with continuous stirring at 100 rpm. The resulting dispersion was diluted 1:100 and analyzed by dynamic light scattering for hydrodynamic diameter, zeta potential, PDI, and SEM. To further analyze the loading capacity of the sMN, the tips were carefully scratched from the backing layer using a scalpel and dissolved in 5 mL of a 1:1 (v/v) mixture of DCM and acetonitrile under vigorous vortexing for 15 min. This process disrupted the PLGA matrix in GLNPs, releasing the GL. After filtration through a 0.45 µm PTFE syringe filter, the concentrations of the released GL were determined. For TS, which is water-soluble, the microneedle tips from TS+GLNPs@sMN were dissolved in ultrapure water, and the TS concentration was determined directly.

***In vitro* anti-proliferative activity**

HS progression is driven by persistent myofibroblast survival; the selective induction of apoptosis in these cells is therefore a validated therapeutic strategy [6]. To evaluate the *in vitro* anti-proliferative activity, we initially assessed the dose-dependent inhibitory effects of free GL, TS, and GLNPs on HSFs. Subsequently, we analyze the anti-proliferative effects of the corresponding formulations, including control, TS, GLNPs, sMN, TS@sMN, GLNPs@sMN, and TS+GLNPs@sMN. HSFs were maintained in DMEM/F-12 containing 10 % FBS and 1 % penicillin–streptomycin at 37 °C, 5 % CO₂, 95 % humidity. For initial screening, 5 × 10^3^ cells per well were seeded in 96-well plates, allowed to attach for 12 h, and exposed for 24 h to serial dilutions of GL (0–50 µM) prepared from a 50 mM stock in 0.1 % (v/v) DMSO (final vehicle ≤ 0.2 %), and TS (0–100 µM) prepared from an aqueous stock (ultrapure water, 0.22 µm filtered). Subsequently, GLNPs were tested at concentrations ranging from 0 to 100 µM. Later, formulations, including sMN, GLNPs@sMN, TS@sMN, and TS+GLNPs@sMN, were tested at equivalent drug concentrations based on the previous results. Cell viability was assessed using the CCK-8 assay (450 nm) at 24 and 48 h.

$$\text{Cell viability (\%)=(}\frac{\text{A}_{\text{exp}}\text{ -}\text{A}_{\text{blank}}}{\text{A}_{\text{ctrl}}\text{-}\text{A}_{\text{blank}}}\text{)×100 }$$

Where A_exp_ denotes the absorbance of the experimental sample, A_blank_ indicates the absorbance of the blank sample, and A_ctrl_ signifies the absorbance of the control sample. Further, after 24 h, the Calcein-AM/PI double-staining kit was used to evaluate cell viability and assess the proportion of live and dead cells within the population.

***In vitro* scratch-wound assay**

HSFs were seeded at 2.0 × 10^5^ cells per well in 6-well plates and cultured in 2 mL of DMEM/F-12 medium supplemented with 10% (v/v) FBS. The cells were maintained at 37°C in a humidified atmosphere containing 5% CO_2_ until they reached 80–90% confluence. A uniform cell-free gap was created by manually scraping the monolayer with a sterile 200 µL pipette tip along a straight axis. Floating cells were removed by gently washing the wells twice with PBS. Subsequently, the medium was replaced with 2 mL of DMEM/F-12 supplemented with 0.5% (v/v) FBS, either containing TS+GLNPs@sMN or without (medium only). Phase-contrast micrographs of identical fields were acquired immediately after wounding (0 h), 18h, and 24 h after wounding. The remaining uncovered area was measured using ImageJ software. The extent of wound closure was quantified using the following equation:

$$\text{Migration rate (\%)=(}\frac{\text{M}_{\text{0}}\text{ - }\text{M}_{\text{t}}}{\text{M}_{\text{0}}}\text{)×100 }$$

Where M_0_ represents the initial wound area at 0 h, and M_t_ represents the wound area at time t (18 h or 24 h).

***In vitro* angiogenesis analysis**

To elucidate the inhibitory effects of TA+GLNPs@sMN on the angiogenesis ability of HSFs, an *in vitro* tube formation assay was conducted. The GelNest™ Matrix (high concentration) was added to a 48-well plate and incubated at 37°C for 30 min to allow gelation. HSFs were then seeded at a density of 5 × 10^4^ cells per well onto the matrix-coated plate and treated with TA+GLNPs@sMN for 6 h. A control group with no treatment (blank) was also included. Capillary-like structures were visualized using an Olympus SZ61 stereomicroscope, and the length of the formed tubes and the number of branch points were quantified using ImageJ software.

**Biocompatibility evaluation of TS+GLNPs@sMN**

Prior to therapeutic application against HS, the local and systemic compatibility of the microneedle formulation (TS+GLNPs@sMN) was evaluated in male C57BL/6 mice. The mice's dorsal hair was removed with depilatory cream 24 h before intervention to ensure complete hair follicle clearance and uniform exposure. Mice were randomly assigned to two groups (n = 6 each), control (sterile PBS, 100 µL topically) or TS+GLNPs@sMN (single application for 4 weeks with gentle thumb pressure for 30 s). Digital photographs were acquired on Day 1 to record any erythema, oedema, epidermal disruption, or skin irritation. Body weights were monitored every 48 h for 28 days as a global indicator of well-being. On Day 28, animals were euthanized under isoflurane anesthesia, and blood samples were collected via cardiac puncture for detailed hematological and biochemical analyses. Complete blood counts (CBCs) were performed to assess overall blood health and identify hematological abnormalities. Kidney function was evaluated with standard renal function tests, and liver function was assessed with a panel of hepatic function tests. These analyses were conducted on both the control group and the TS+GLNPs@sMN patch-treated group to determine the impact of the treatment on these physiological parameters. The treated dorsal skin and major organs (heart, liver, kidneys, spleen, and small intestine) were also excised, rinsed in ice-cold saline, and fixed in 4 % (w/v) neutral-buffered formalin for 24 h. After progressive dehydration via graded ethanol, tissues were embedded in paraffin, sectioned at 4–5 µm, and stained with routine H&E. The stained sections were examined under a light microscope to assess for signs of inflammation, necrosis, or structural alterations.

**Histological and biochemical assay**

Treated skin samples, intended for histopathological and immunohistochemical analyses, were fixed in 4% (w/v) paraformaldehyde for 24 h, dehydrated through a graded ethanol series, cleared in xylene, and embedded in paraffin. Serial sections (4 µm) were prepared and subjected to multiple staining protocols to assess various histological parameters. H&E staining was used to evaluate general tissue morphology, Scar Elevation Index (SEI), and Epidermal Thickness Index (ETI). Masson’s trichrome staining was employed to visualize overall collagen deposition and distribution. The SEI was calculated using the formula: SEI = D_HS_/D_NS_, where D_HS_ represents the vertical distance from the highest point of the HS to the cartilage surface, and D_NS_ denotes the corresponding distance in normal skin from the stratum corneum to the cartilage surface. The ETI was calculated by dividing the mean epidermal thickness in HS tissues by that in normal skin, as assessed from H&E-stained sections. An ETI value above 1 is usually a marker of epidermal hypertrophy. To determine the collagen-I to collagen-III ratio, we applied Sirius red staining, a recognized technique for assessing collagen fiber organization and distinguishing collagen-I from collagen-III. Under polarized light, thick collagen-I fibers appear yellowish-orange to red, while thin collagen-III fibers show green to yellowish-green against a dark background. Collagen-I is vital for forming the extracellular matrix scaffold, and collagen-III affects fiber diameter and elasticity. The ratio of collagen-I to collagen-III is key to wound healing, as an imbalance can delay healing and promote scarring. Higher collagen-III levels are associated with reduced scarring. Following staining procedures, tissue sections were meticulously examined and photographed under a NIKON Eclipse ci polarizing microscope with a DS-FI2 camera and NIS Elements software to assess collagen fiber properties. Further, immunohistochemical staining was performed to detect TGF-β1 and α-SMA, markers of profibrotic signaling and myofibroblast activation, respectively. Additionally, TGF-β1 levels were quantified using an enzyme-linked immunosorbent assay (ELISA), and hydroxyproline (HYP) levels were determined by a colorimetric assay. The concentrations of VEGF, TNF-α, IL-6, and tryptase were measured using a dual-antibody sandwich ELISA, following the manufacturer's instructions.

**Figures S1 to S9**

**
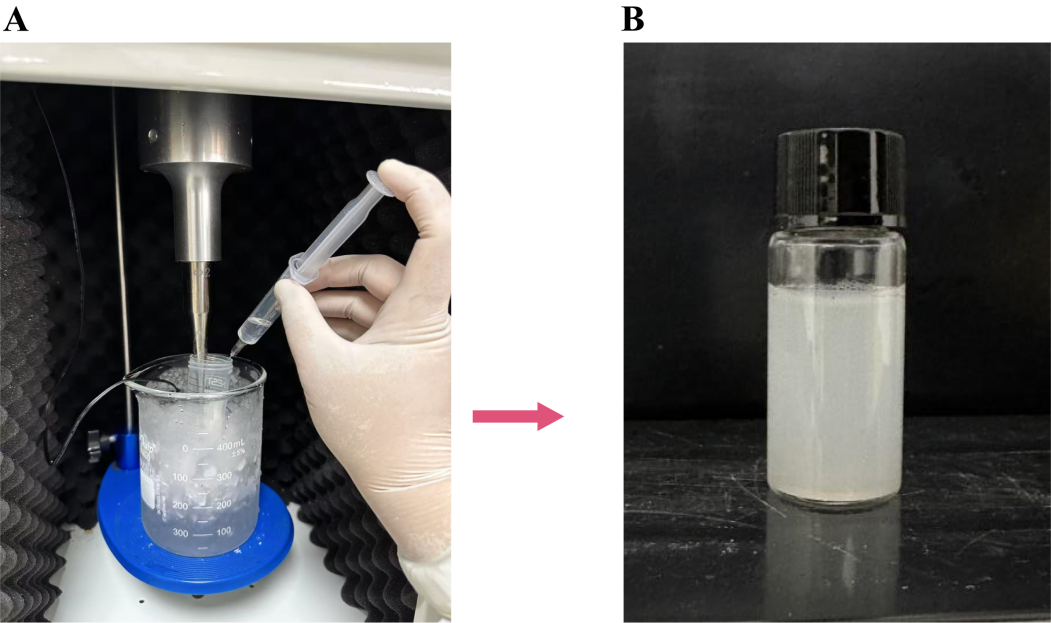
**

**Figure S1:** (A) Preparation of o/w nanoemulsion (B) GLNPs nanoemulsion.


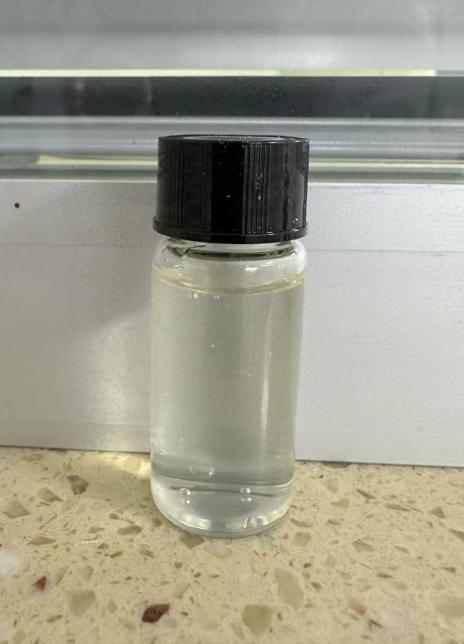


**Figure S2:** Photograph of a 5% sodium alginate hydrogel in a glass vial


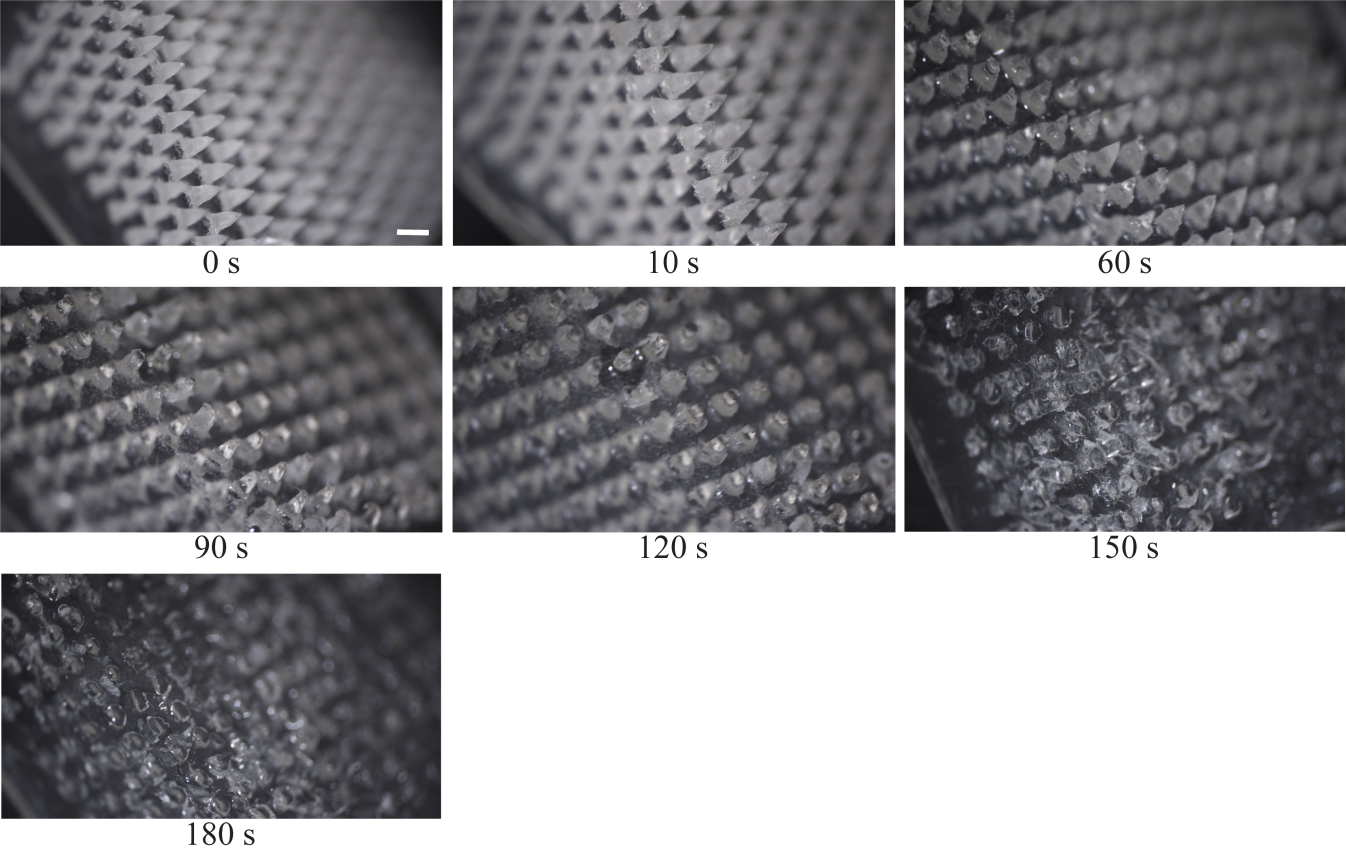


**Figure S3:** Sequential dissolution of sMN in rabbit ear HS model showing tip bending, softening, and progressive dissolution without fracture (Scale bar: 800 μm).

**
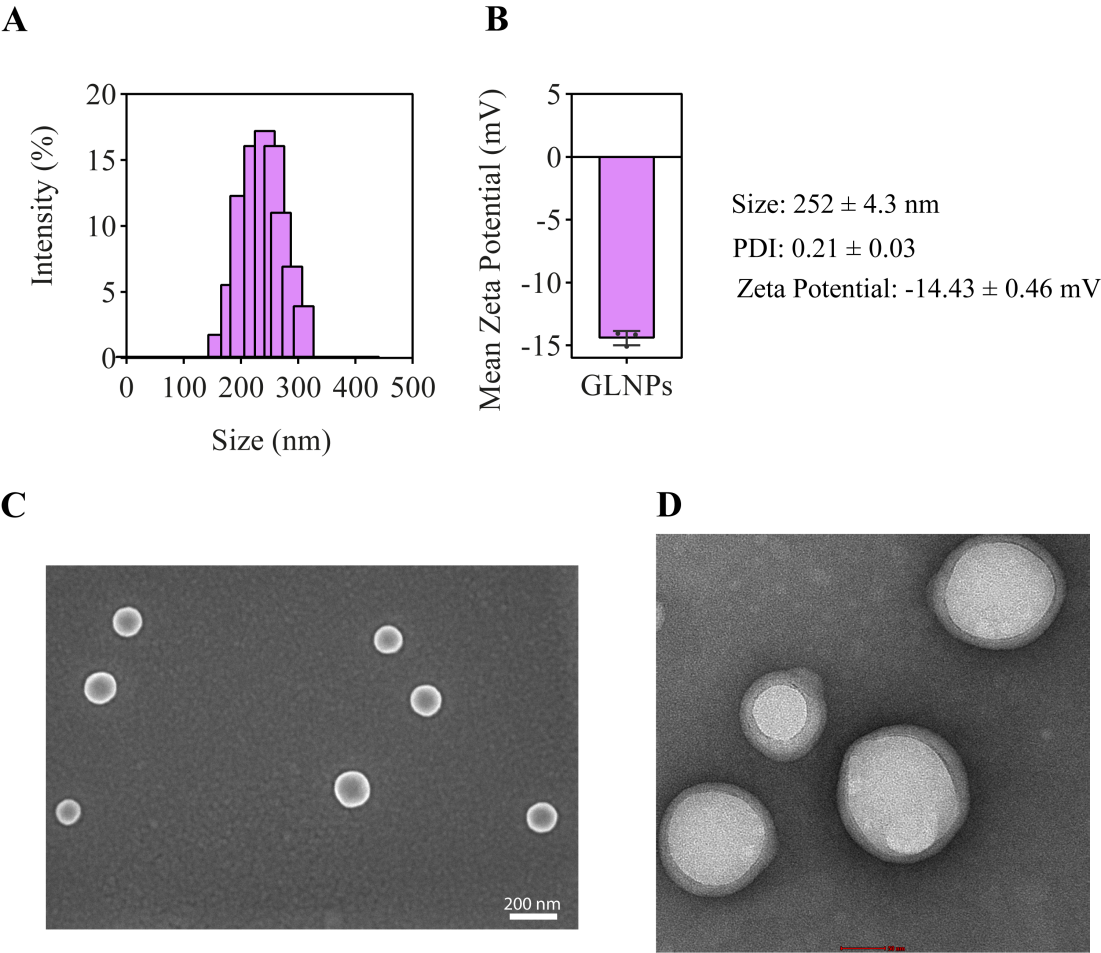
**

**Figure S4:** (A) Particle size and (B) Zeta potential of GLNPs recovered from dissolved microneedle tips. (C) SEM (Scale bar: 200 nm) and (D) TEM images depicting GLNPs morphology and core-shell architecture after incorporation into sMN (Scale bar: 50 nm).

**
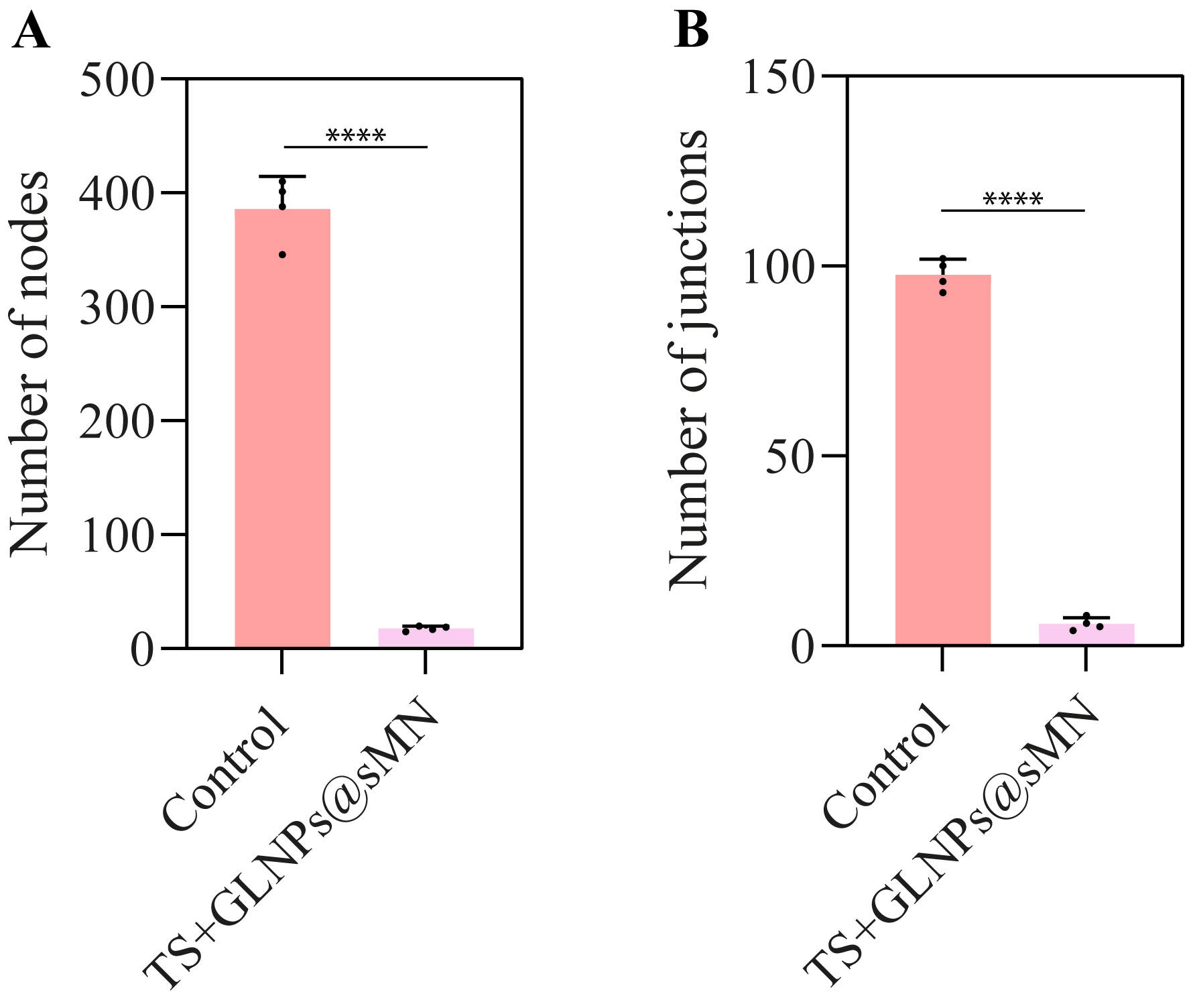
**

**Figure S5:** (A) Quantification of tubule nodes by ImageJ. (B) Quantification of tubule junctions by ImageJ (n=4, mean ± SD, *p < 0.05, **p < 0.01, ***p < 0.001, and **** p < 0.0001).

**
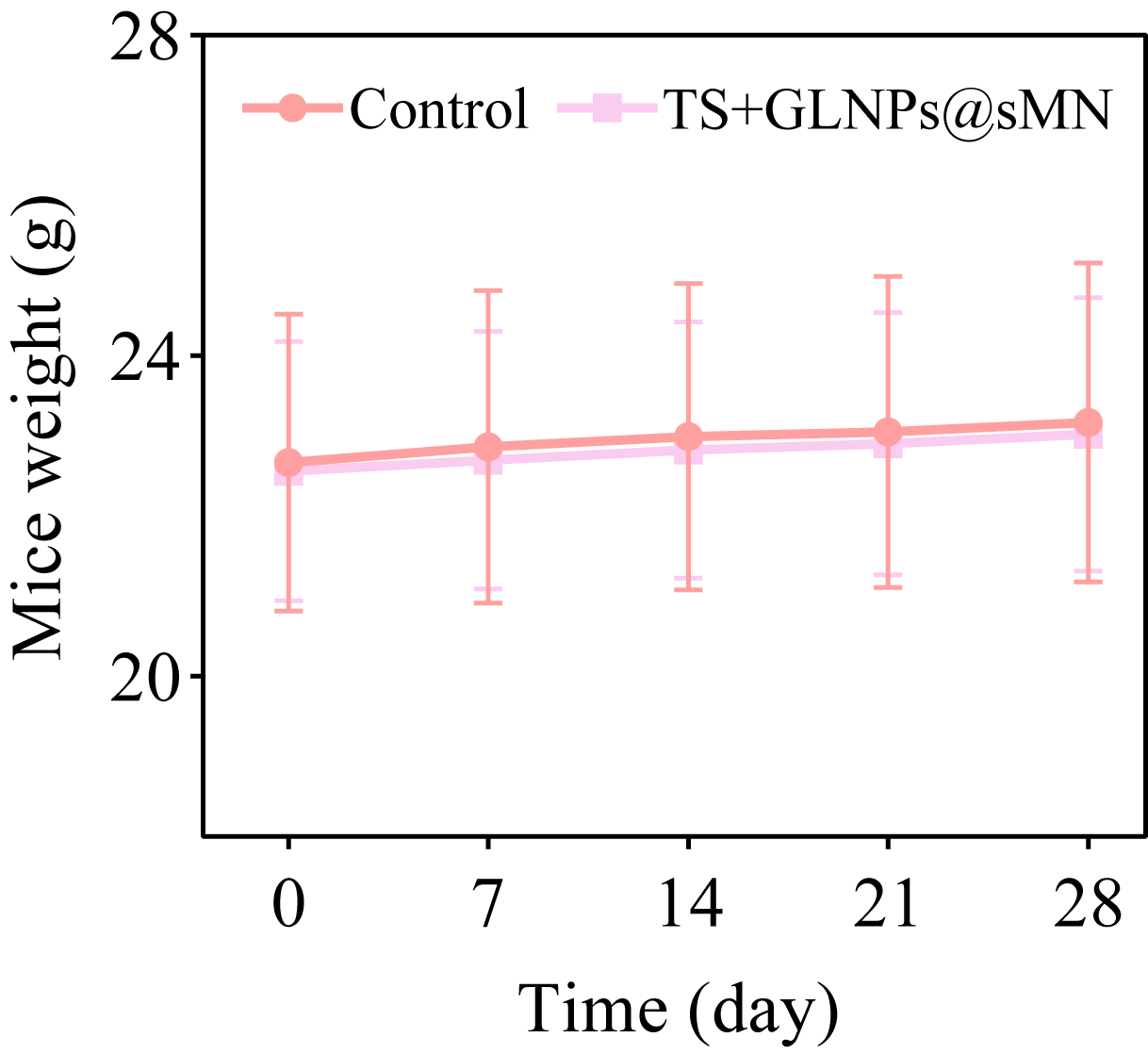
**

**Figure S6:** Mice weight after application of control (Normal) and TS+GLNPs@sMN (n=3).


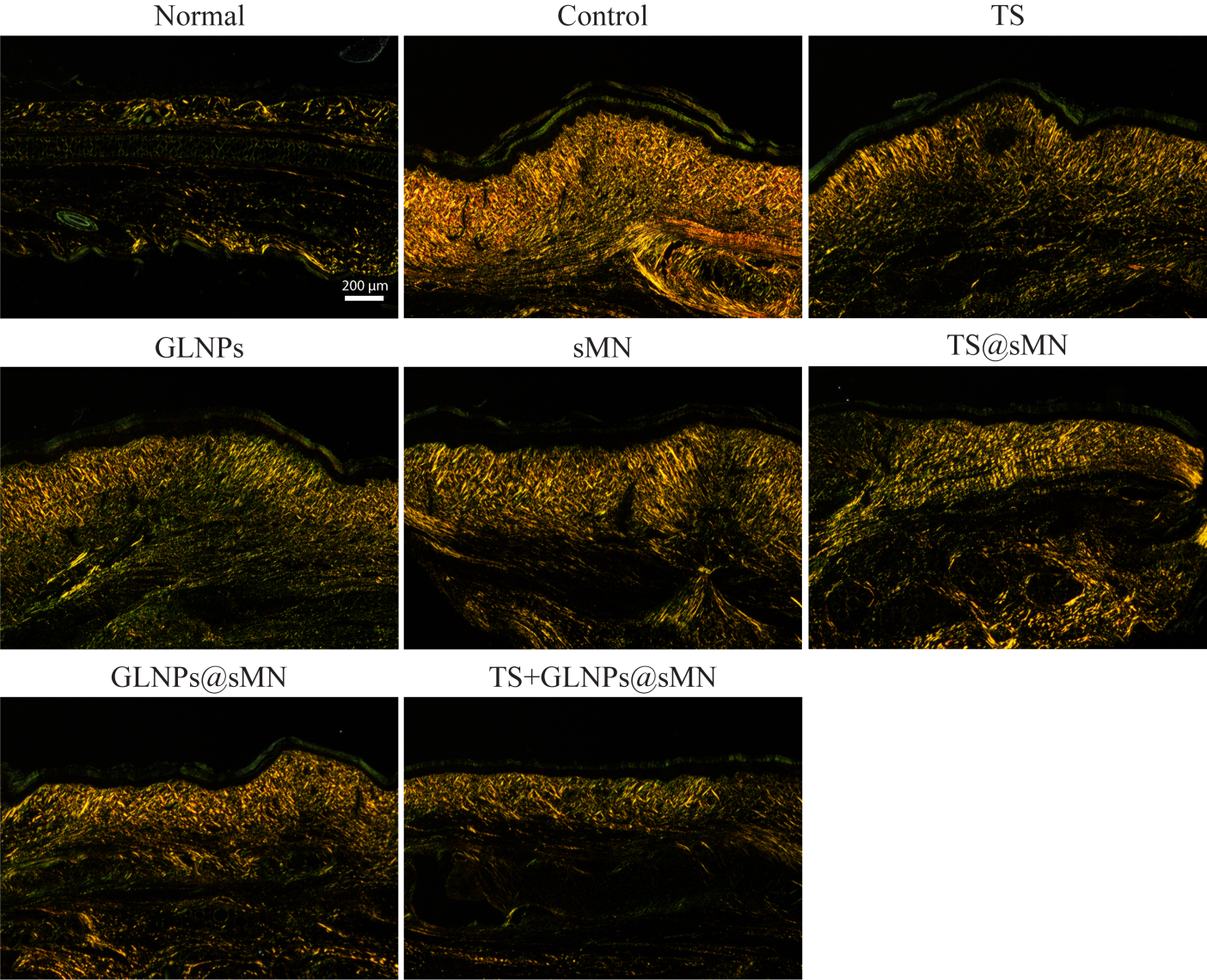


**Figure S7**: Large-area polarized light microscopy images of Sirius red-stained sections showing collagen fiber distribution across treatment groups (Scale bar: 200 μm).


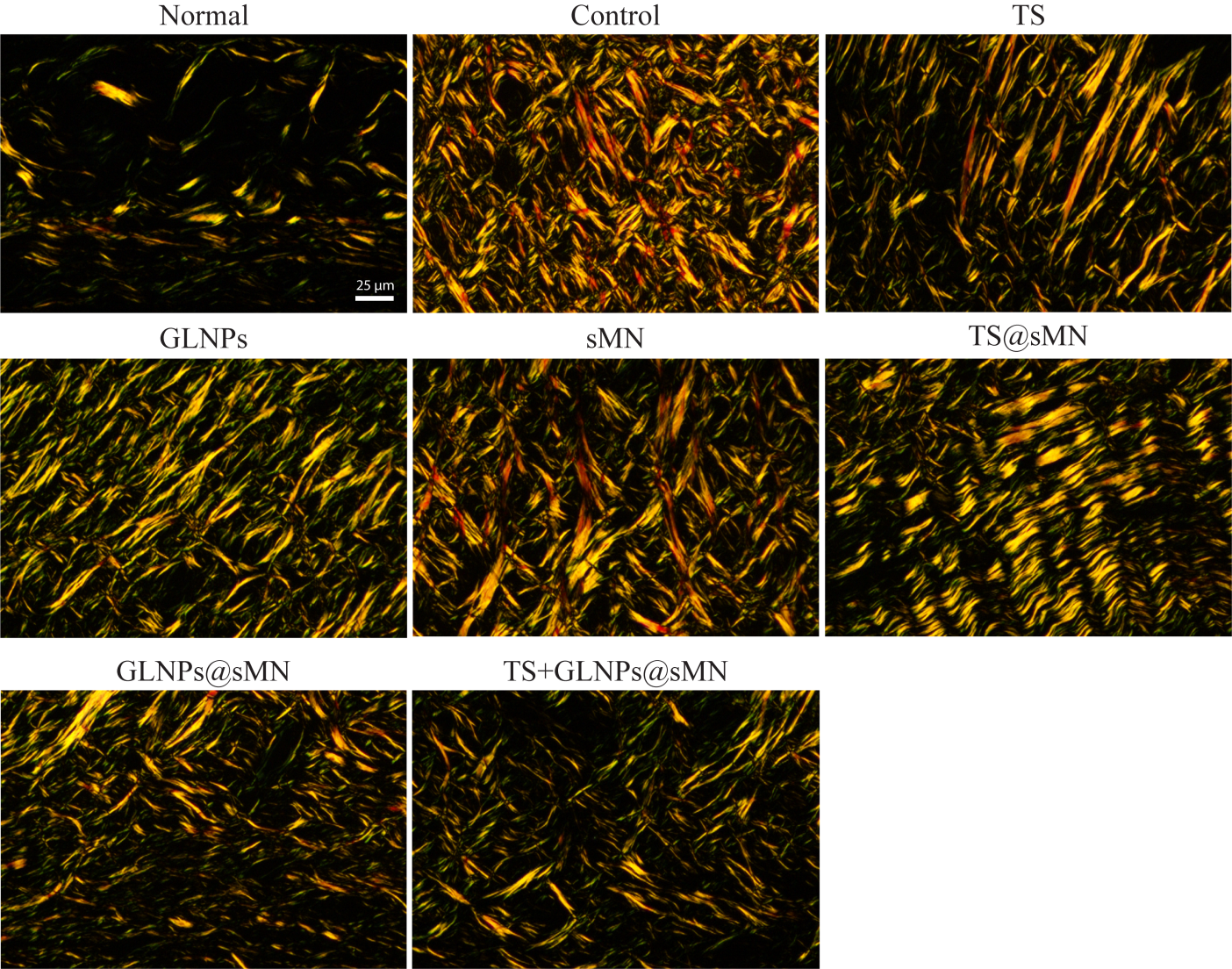


**Figure S8**: High-magnification polarized light microscopy images of Sirius red-stained sections, illustrating detailed collagen fiber morphology across different treatment groups (Scale bar: 25 μm).


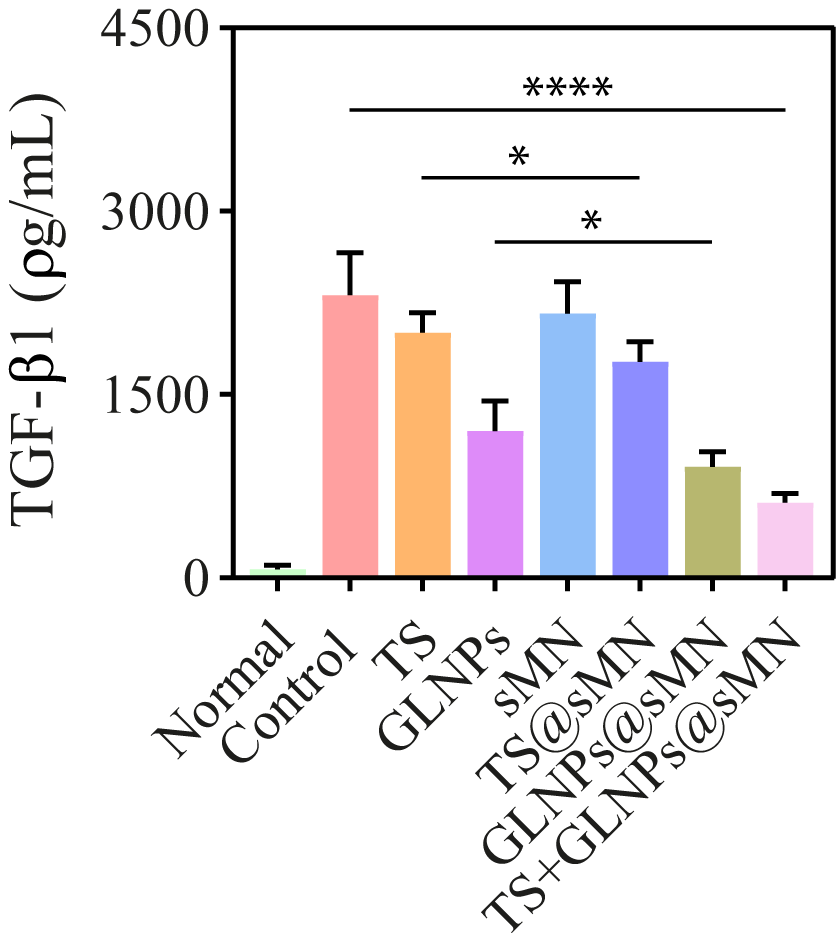


**Figure S9**: Quantification of TGF-β1 levels in skin samples treated with various formulations (n=6, mean ± SD, *p < 0.05, **p < 0.01, ***p < 0.001, and **** p < 0.0001).

**Table S1**

**Table S1:** The weight of the microneedle patch (sMN), drug-loading capacity, and physicochemical properties (size, PDI, zeta potential) of GLNPs embedded in the microneedles (n=3, mean ± SD).

| **Formulation/ Component** | **Weight (mg)** | | **sMN array loading capacity** | **Diameter (nm)** | **PDI** | **Zeta Potential (mV)** |
| --- | --- | --- | --- | --- | --- | --- |
|  | Microneedles | Base layer |  |  |  |  |
| **sMN** | 5.6 ± 0.4 | 10.09 ± 0.61 | - | - | - | - |
| **TS** | - | - | 0.031 ± 0.002 mg | - | - | - |
| **GL** | - | - | 0.023 ± 0.004 mg | - | - | - |
| **GLNPs** | - | - | - | 252 ± 4.3 | 0.21 ± 0.03 | -14.43 ± 0.46 |
